# Repurposing Tamoxifen as Potential Host-Directed Therapeutic for Tuberculosis

**DOI:** 10.1128/mbio.03024-22

**Published:** 2022-12-07

**Authors:** Ralf Boland, Matthias T. Heemskerk, Gabriel Forn-Cuní, Cornelis J. Korbee, Kimberley V. Walburg, Jeroen J. Esselink, Carina Carvalho dos Santos, Amy M. de Waal, Daniel C. M. van der Hoeven, Elisa van der Sar, Alex S. de Ries, Jiajun Xie, Herman P. Spaink, Michiel van der Vaart, Mariëlle C. Haks, Annemarie H. Meijer, Tom H. M. Ottenhoff

**Affiliations:** a Institute of Biology Leiden, Leiden University, Leiden, The Netherlands; b Department of Infectious Diseases, Leiden University Medical Center, Leiden, The Netherlands; c Laboratório Especial de Desenvolvimento de Vacinas, Instituto Butantan, São Paulo, Brazil; Institut Pasteur

**Keywords:** tuberculosis, tamoxifen, HDT, lysosomal acidification, human macrophages, zebrafish model, MDR, AMR, host-directed therapeutic

## Abstract

The global burden of tuberculosis (TB) is aggravated by the continuously increasing emergence of drug resistance, highlighting the need for innovative therapeutic options. The concept of host-directed therapy (HDT) as adjunctive to classical antibacterial therapy with antibiotics represents a novel and promising approach for treating TB. Here, we have focused on repurposing the clinically used anticancer drug tamoxifen, which was identified as a molecule with strong host-directed activity against intracellular Mycobacterium tuberculosis (*Mtb*). Using a primary human macrophage *Mtb* infection model, we demonstrate the potential of tamoxifen against drug-sensitive as well as drug-resistant *Mtb* bacteria. The therapeutic effect of tamoxifen was confirmed in an *in vivo* TB model based on Mycobacterium marinum infection of zebrafish larvae. Tamoxifen had no direct antimicrobial effects at the concentrations used, confirming that tamoxifen acted as an HDT drug. Furthermore, we demonstrate that the antimycobacterial effect of tamoxifen is independent of its well-known target the estrogen receptor (ER) pathway, but instead acts by modulating autophagy, in particular the lysosomal pathway. Through RNA sequencing and microscopic colocalization studies, we show that tamoxifen stimulates lysosomal activation and increases the localization of mycobacteria in lysosomes both *in vitro* and *in vivo*, while inhibition of lysosomal activity during tamoxifen treatment partly restores mycobacterial survival. Thus, our work highlights the HDT potential of tamoxifen and proposes it as a repurposed molecule for the treatment of TB.

## INTRODUCTION

It is estimated that 1.7 billion people are latently infected with Mycobacterium tuberculosis (*Mtb*), the infectious agent causing tuberculosis (TB) (1). In 2020, there were 10 million new cases, and 1.4 million people died from the disease (2). There is an alarming contribution of multidrug-resistant (MDR-TB) and extensively drug-resistant (XDR-TB) infections to the global antimicrobial resistance (AMR) disease burden (2). Currently, there is no effective TB vaccine available, and the only licensed vaccine in use, Bacille Calmette-Guérin (BCG), has limited protective efficacy (3). Although in the last decade, a few new antibiotics have been approved for the treatment of MDR- and XDR-TB, including bedaquiline (4), delamanid (5), linezolid (6), and pretomanid (7), mutations conferring resistance against these drugs have already been found (8). Therefore, novel tools and strategies are needed to combat this global threat, including more effective therapeutics that shorten the prolonged regimens of TB treatment (currently 6 months or more) and help prevent *de novo* resistance and TB relapse.

Intracellular bacteria such as *Mtb* manipulate cellular signaling pathways to promote their own survival in human cells by creating a replicative niche or by subverting the immune system (9, 10). As a complement to classical antibiotics, host-directed therapy (HDT) has recently emerged as a novel concept in TB. HDT aims to enhance host defense by modulating processes in the host that restrict growth and survival of bacteria in their intracellular niches (11–15). Large-scale chemical and genetic screens of molecular libraries targeting *Mtb*-infected cells have revealed a variety of potential HDT candidates that could be repurposed to combat TB, including groups of anti-inflammatory drugs, antipsychotic drugs, and kinase inhibitors. These compounds affecting inflammatory pathways, lipid metabolism, and autophagy could be effective against both antibiotic-sensitive and antibiotic-resistant bacteria, including MDR- and XDR-TB (11–15).

Autophagy is an intracellular degradation pathway vital to maintaining homeostasis that removes unwanted elements from the cytosol, such as misfolded protein aggregates, damaged organelles, and microbial invaders (16). Due to the prohomeostatic function of autophagy, drugs that modulate this process are currently being investigated as novel therapeutics for a wide variety of diseases (17). Autophagy can inhibit intracellular infection by promoting the delivery of pathogens to lysosomes (18). Although virulence mechanisms of pathogens may counteract autophagy to different extents (19), several studies have shown that induction of autophagy restricts *Mtb* intracellular growth and promotes its lysosomal degradation (16, 20, 21). For these reasons, autophagy has become a priority target for anti(myco)bacterial HDT development (13, 15, 18, 22).

Tamoxifen, widely known for its use as a breast cancer therapeutic (23–25), was identified as a promising molecule for host-directed inhibition of intracellular *Mtb* when we previously screened an autophagy-modulating compound library *in vitro* in human cells (26). The main known targets of tamoxifen are estrogen receptors (ERs). Tamoxifen can function either as an agonist or antagonist of the ER, depending on the presence of coregulatory transcription factors (24). Besides its use in breast cancer therapy or in biomedical research for ER-based inducible gene expression systems, tamoxifen has more recently been studied in the context of various microbial infections and was found to possess direct antimicrobial effects against Cryptococcus and *Leishmania* (27, 28). In addition, it was reported that tamoxifen had a direct antibacterial effect on *Mtb*, synergizing with first-line TB antibiotics (29, 30). In contrast to these reported direct antimicrobial effects, there is evidence that the inhibitory effect of tamoxifen on intracellular *Toxoplasma* growth is mediated in a host-directed manner by inducing autophagic degradation of the parasite-containing vacuole (31). However, the role of tamoxifen-induced autophagy and possibly other tamoxifen-modulated host pathways in controlling *Mtb* or other bacterial infections remains incompletely defined.

In this study, we have used *in vitro* and *in vivo* TB models to investigate the antibacterial and host-directed effects of tamoxifen and to elucidate the potential host-directed mechanisms involved. Lung-resident macrophages, consisting mainly of alveolar macrophages, represent the predominant host cell in the initial stages of *Mtb* infection (32, 33). The different functional responses of these cells can be represented by differentiating primary human macrophages *in vitro* into pro- and anti-inflammatory polarization states (34), which proved an effective approach to explore drug efficacy (35–37). To investigate the *in vivo* therapeutic potential of tamoxifen, we used the zebrafish TB model, which reiterates many features of human TB pathogenesis (38–40). Specifically, infection of zebrafish embryos with Mycobacterium marinum (*Mm*), which shares major virulence factors with *Mtb*, results in the development of granulomatous aggregates of leukocytes, the hallmark pathology of TB. Moreover, we have previously demonstrated that autophagy is a critical host defense mechanism of zebrafish to *Mm* infection, which makes this model well suited to investigate the autophagy-modulating properties of tamoxifen in relation to mycobacterial pathogenesis (41–44).

Using *in vitro* infected human macrophages, we demonstrate a clear HDT effect of tamoxifen against both drug-susceptible and MDR-*Mtb* strains. Furthermore, we found that tamoxifen’s HDT effect against intracellular mycobacteria is independent of ER signaling both *in vitro* and *in vivo*. Complementary transcriptome profiling of zebrafish larvae revealed significant effects of tamoxifen on pathways related to autophagy and lysosomal processes, both in the absence and presence of infection. Colocalization analyses of *Mtb* and *Mm* with autophagosomal and lysosomal markers showed that the HDT effect of tamoxifen could not be directly attributed to its autophagy-inducing properties but appears linked to modulation of lysosomal function or increased delivery of mycobacteria to lysosomes. Indeed, the addition of bafilomycin, a vacuolar-type ATPase (V-ATPase) inhibitor that abrogates lysosomal activity, partly inhibited the effect of tamoxifen on mycobacterial survival. In conclusion, our results suggest that tamoxifen inhibits intracellular mycobacteria primarily by promoting the efficacy of the lysosomal pathway, which was cross-validated across different hosts and different mycobacterial pathogens. Our findings position this clinically approved drug as a strong candidate for repurposing as an HDT molecule against TB, especially MDR- and XDR-TB.

## RESULTS

### *In vitro* identification of tamoxifen as a novel repurposed host-directed therapeutic.

**FIG 1:**
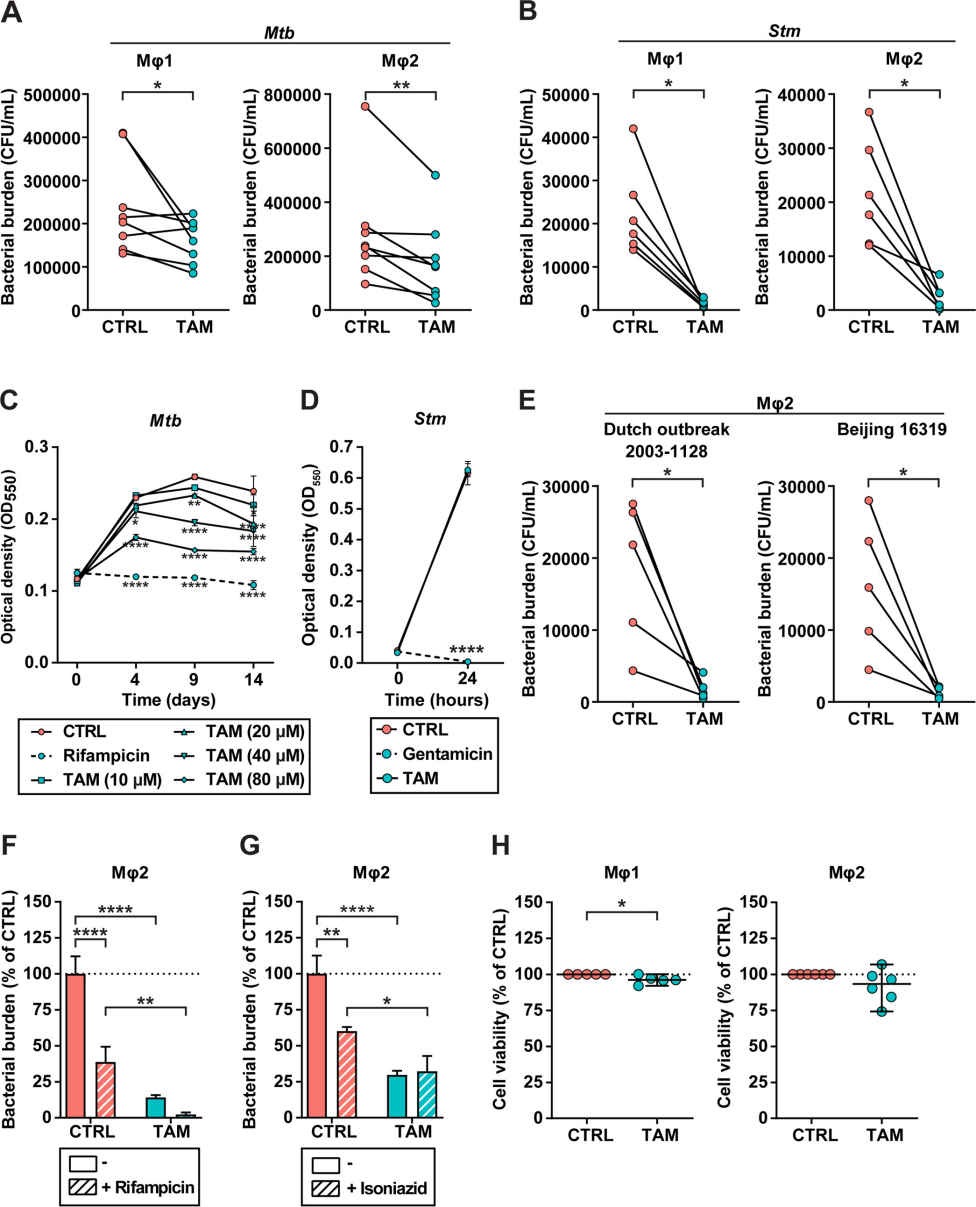
*In vitro* identification of tamoxifen as a novel repurposed host-directed therapeutic. (A and B) CFU assay of Mφ1 (left) and Mφ2 (right) macrophages infected with H37Rv-*Mtb* (A) or *Stm* (B) and treated with 10 μM tamoxifen (TAM) or control (CTRL; DMSO at equal volume) for 24 h. Each dot represents a single donor (8 and 9 donors for Mφ1 and Mφ2 macrophages, respectively, in A, and 6 donors in B) and depicts the mean of 3 or 4 replicates. Statistical significance was tested using by Wilcoxon matched-pairs signed-rank test. (C and D) H37Rv-*Mtb* growth (C) or *Stm* growth (D) in liquid culture during treatment with 10, 20, 40, or 80 μM tamoxifen or control (DMSO at equal volume [10 μM is shown]) up to the assay endpoint day 14 (C) or overnight (D). Rifampicin (20 μg/mL) (C) or gentamicin (50 μg/mL) (D) was used as a positive control for growth inhibition. Each line depicts the mean ± standard deviation of three replicates. The experiment shown is a representative of two (C) or three (D) independent experiments. Statistical significance of treatment versus control treatment was tested by two-way ANOVA with Dunnett’s multiple-comparison test. (E) CFU assay of Mφ2 macrophages infected with MDR-*Mtb* strain Dutch outbreak 2003-1128 (left) or *Mtb* Beijing strain 16319 (right) and treated with 10 μM tamoxifen or control (DMSO at equal volume) for 24 h. Each dot represents a single donor (6 donors in total) and depicts the mean of three replicates. Statistical significance was tested using a ratio paired *t* test. (F and G) CFU assay of Mφ2 macrophages infected with H37Rv-*Mtb* and treated for 24 h with 10 μM tamoxifen or control (DMSO at equal volume) in combination with suboptimal doses of 0.05 μg/mL rifampicin (F) or 0.4 μg/mL isoniazid (G). Each bar depicts the mean ± standard deviation of three replicates from a representative donor (out of 4 donors tested in F and 3 donors in G) expressed as a percentage of the control treatment in the absence of antibiotics. Bars with solid colors represent tamoxifen or control treatment only, and bars with patterns represent the combination with antibiotic. Statistical significance was tested by two-way ANOVA with Tukey’s multiple-comparison test comparing tamoxifen treatment (in the absence or presence of antibiotics) to the corresponding control treatment. (H) LDH release assay of Mφ1 (left) and Mφ2 (right) macrophages infected with H37Rv-*Mtb* and treated with 10 μM tamoxifen or control (DMSO at equal volume) for 24 h. Each dot represents a single donor (5 and 6 donors for Mφ1 and Mφ2 macrophages, respectively) and depicts the mean of three replicates. Dotted lines indicate the control set at 100%, and median values + 95% confidence intervals are shown for every condition. Statistical significance was tested using a ratio paired *t* test; *, *P* < 0.05; **, *P* < 0.01; ***, *P* < 0.001; ****, *P* < 0.0001.

For *de novo* discovery of drugs with potential activity against intracellular *Mtb*, we previously screened the Screen-Well autophagy library of clinically approved molecules by treating infected cells for 24 h, which identified tamoxifen as a promising candidate (26). We purposely applied a short treatment duration to identify the most potent drugs only. To validate this initial screening result, we tested tamoxifen in our previously described primary human macrophage model system. We compared tamoxifen’s effects on intracellular infection in two polarized macrophage subsets, proinflammatory (Mφ1) and anti-inflammatory (Mφ2) macrophages (35, 45, 46). Classical CFU assays were used to measure the effect of 24-h treatment on *Mtb* infection (Fig. 1A; Table S1 in the supplemental material). Tamoxifen treatment showed a significant decrease of *Mtb* outgrowth in both Mφ1 and Mφ2 macrophages (median reduction of detectable bacteria of 29% and 44%, respectively). To test whether tamoxifen could also target another intracellular pathogen, we infected Mφ1 and Mφ2 macrophages with Salmonella enterica serovar Typhimurium (*Stm*) (Fig. 1B; Table S1). Tamoxifen showed high efficacy against intracellular *Stm* outgrowth (in several donors, we observed up to 99% reduction of detectable bacteria).

10.1128/mbio.03024-22.6 TABLE S1 CFU data of Mφ1 and Mφ2 macrophages corresponding to Fig. 1. Download Table S1, PDF file, 0.4 MB. Copyright © 2022 Boland et al. 2022 Boland et al. https://creativecommons.org/licenses/by/4.0/ This content is distributed under the terms of the Creative Commons Attribution 4.0 International license.

To confirm that tamoxifen acts in a host-directed and not direct antibacterial manner, we treated both *Mtb* and *Stm* in liquid broth with tamoxifen at the same concentration (10 μM). *Mtb* growth was only affected by the presence of 10 μM tamoxifen after the culture was in stationary phase, whereas the positive-control anti-*Mtb* antibiotic rifampicin inhibited *Mtb* growth as expected (Fig. 1C). Higher doses of tamoxifen did impact *Mtb* growth as reported previously (27–30). Tamoxifen treatment at a concentration of 10 μM did not affect *Stm* growth, while the control anti-*Stm* antibiotic gentamicin completely prevented bacterial proliferation (Fig. 1D).

Host-directed drugs are expected to work irrespective of the exact mycobacterial substrain targeted, including drug-susceptible and multidrug-resistant (MDR) *Mtb* strains. Because tamoxifen demonstrated similar efficacy in both Mφ1 and Mφ2 macrophages (no significant difference was found when comparing the responses in Mφ1 versus Mφ2 macrophages to tamoxifen using a paired *t* test), we decided to focus further on Mφ2 macrophages. Tamoxifen treatment of Mφ2 macrophages infected with two MDR-*Mtb* strains (*Mtb* Dutch outbreak strain 2003-1128 and *Mtb* Beijing strain 16319) significantly inhibited bacterial outgrowth in both cases (Fig. 1E).

Additionally, because HDT molecules and classical antibiotics by definition target different molecules, positive interactivity might be anticipated during combined treatment. Indeed, tamoxifen combined with a suboptimal dose of rifampicin (0.05 μg/mL) inhibited bacterial outgrowth more effectively than either molecule individually (Fig. 1F). However, this effect was not observed when tamoxifen was combined with a suboptimal dose of isoniazid (0.4 μg/mL) (Fig. 1G), suggesting that the effect of interactivity depends on the particular combination of tamoxifen with antibiotics. Lastly, tamoxifen treatment showed no toxicity toward Mφ1 and Mφ2 macrophages (Fig. 1H) (although a significant difference was found in Mφ1 macrophagess, this difference was deemed biologically irrelevant because the average reduction was 3.8%).

Taken together, we report strong HDT activity of tamoxifen against both intracellular *Mtb* and *Stm* in primary human macrophages regardless of their M1 or M2 polarization state. Furthermore, we demonstrate that tamoxifen shows efficacy against both drug-sensitive-*Mtb* (DS-*Mtb*) and MDR-bacteria (*Mtb*) and that it might be used as a safe adjunctive to classical antibiotics such as rifampicin.

### *In vivo* validation of tamoxifen as an HDT.

**FIG 2:**
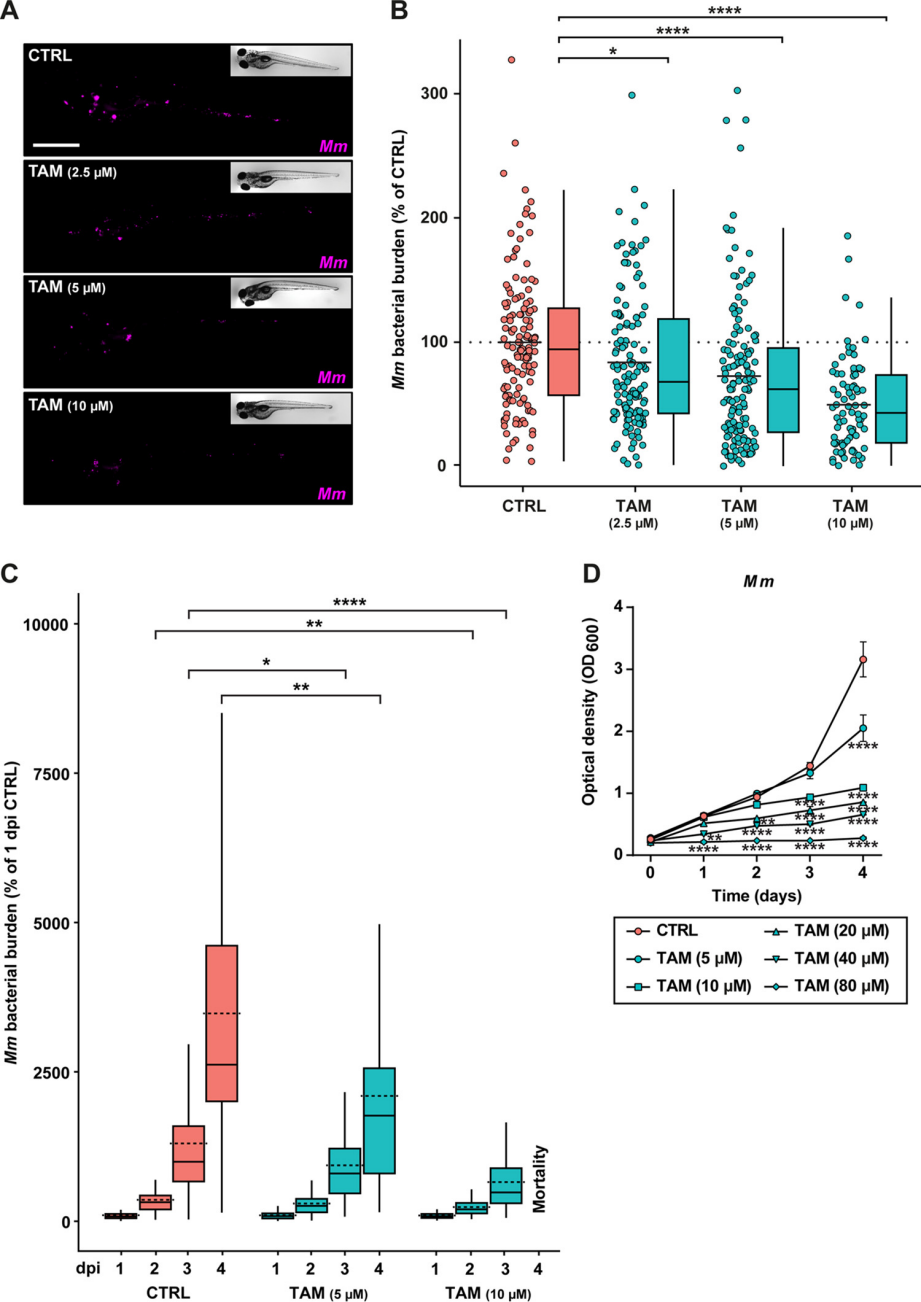
*In vivo* validation of tamoxifen as an HDT. (A) Bacterial burden assay of mWasabi-expressing *Mm*-infected zebrafish larvae treated with increasing doses of tamoxifen (2.5, 5, and 10 μM) or control (DMSO at 0.1% [vol/vol]). Treatment was started at 1 hpi, and larvae were anesthetized at 4 dpi for imaging. Representative stereo fluorescent images of whole larvae infected with mWasabi-expressing *Mm* are shown. Magenta shows *Mm*; scale bar, 1 mm. (B) Quantification of bacterial burden shown in A. Bacterial burden was normalized to the mean of the control group (set at 100% and indicated with the dotted line). Data from 4 experimental repeats were combined (*n* = 132 to 139 per group). Each dot represents a single larva. Box plots with 95% confidence intervals are shown. The black line in the box plots indicates the group median, while the black line in the dot plot indicates the group mean. Statistical analysis was performed using a Kruskal-Wallis test with Dunn’s multiple-comparison test. (C) Bacterial burden assay of mWasabi-expressing *Mm*-infected zebrafish larvae treated with 5 and 10 μM tamoxifen or control (DMSO at 0.1% [vol/vol]). Treatment was started at 1 hpi, and larvae were anesthetized at 1, 2, 3, and 4 dpi for imaging. Bacterial burden was normalized to the control (DMSO at 1 dpi), and data of two experimental repeats were combined (*n* = 65 to 70 per group). All larvae in the 10 μM group died between 3 and 4 dpi. Box plots with 95% confidence intervals are shown. The black line in the box plots indicates the group median, while the dotted line indicates the group mean. Statistical analysis was performed between treatment groups per time point using a Kruskal-Wallis test with Dunn’s multiple-comparison test. (D) *Mm* growth in liquid culture during treatment with 5 or 10 μM tamoxifen or control (DMSO at equal volume) up to the assay endpoint, day 2. Lines depict mean ± standard deviation of two experiments. Statistical significance of treatment versus control treatment was tested using a two-way ANOVA with Dunnett’s multiple-comparison test; *, *P* < 0.05; **, *P* < 0.01; ***, *P* < 0.001; ****, *P* < 0.0001.

To investigate the efficacy of tamoxifen *in vivo*, we used the well-characterized zebrafish TB model, in which embryos are infected with their natural pathogen Mycobacterium marinum. We first validated tamoxifen’s efficacy on *Mm* using the same flow cytometry-based assay as used in the initial screen of HDT candidates for *Mtb*. As anticipated, tamoxifen reduced *Mm* burden in human cells (Fig. S1). Next, we infected zebrafish embryos with mWasabi-labeled *Mm* and treated the infected embryos for 4 days with an increasing dose (2.5, 5, and 10 μM) of tamoxifen or with vehicle (dimethyl sulfoxide [DMSO]) control. Treatment with the highest dose (10 μM) resulted in developmental toxicity (e.g., edema and lethality) in one-third of the larvae, while no toxicity was observed at the lower doses. Bacterial burden was assessed by quantifying the bacterial fluorescent signal of infected larvae at 4 days postinfection (dpi). All doses of tamoxifen treatment reduced bacterial burden significantly compared to the control treatment in a dose-dependent manner (Fig. 2A and B).

10.1128/mbio.03024-22.2 FIG S1 Tamoxifen inhibits *Mm* burden in an *in vitro* infection model. (A) Flow cytometric dot plots of mCherry-expressing *Mm*-infected MelJuSo cells treated with 10 μM tamoxifen or control (DMSO at equal volume) for 24 h. Dot plots consist of 3 concatenated replicates, and the experiment shown is a representative of 2 independent experiments. (B) Quantification of the infected population shown in A. Each bar depicts the mean with standard deviation of 3 replicates. Statistical significance was tested with an unpaired *t* test; **, *P* < 0.01. Download FIG S1, TIF file, 1.3 MB. Copyright © 2022 Boland et al. 2022 Boland et al. https://creativecommons.org/licenses/by/4.0/ This content is distributed under the terms of the Creative Commons Attribution 4.0 International license.

Next, we investigated the infection dynamics during treatment. We infected and treated embryos with tamoxifen (5 and 10 μM) and imaged them daily for 4 days to monitor the establishing infection. Tamoxifen treatment reduced bacterial burden compared to the control treatment from 2 dpi onward, although it was not able to completely block the progression of infection (Fig. 2C). While drug-induced mortality was observed in the 10 μM group at the experimental endpoint (4 dpi), 5 μM tamoxifen treatment resulted in an approximately 2-fold lower infection burden than the control treatment (DMSO) at 4 dpi (Fig. 2C). Nonetheless, we observed that the infection burden increased between 2 and 4 dpi irrespective of treatment, indicating that tamoxifen limits, but does not fully inhibit, bacterial growth.

Although we had found tamoxifen to work as an HDT on *Mtb*-infected cells *in vitro*, we sought to exclude that its effect in the zebrafish TB model was due to a direct antibacterial reduction of *Mm* growth as opposed to a host-directed effect. Therefore, we added tamoxifen at doses of 5, 10, 20, 40, and 80 μM to a liquid culture of *Mm* and assessed bacterial replication by measuring optical density at 600 nm (OD_600_) at five time points from 6 h to the experimental endpoint (4 days). Tamoxifen treatment at a 5 μM concentration only impacted *Mm* significantly after 4 days of culture, while higher doses affected *Mm* growth after 3 days or earlier (Fig. 2D). This supports that the observed reduction of bacterial burden in zebrafish larvae after 3 days of 5 μM tamoxifen treatment and 2 days of 10 μM tamoxifen treatment must be primarily due to a host-directed effect and not a direct antibacterial effect of tamoxifen. For further experiments in our *in vivo* TB model, we used tamoxifen at 5 μM, as this dose consistently lowered bacterial burden in zebrafish larvae in a host-directed manner without causing developmental toxicity.

### Tamoxifen alters leukocyte-specific gene expression without affecting macrophage or neutrophil migration *in vivo*.

We decided to use the zebrafish TB model to investigate the host transcriptomic response to tamoxifen treatment at a systemic level to obtain mechanistic insight into the observed inhibition of mycobacterial infection. Using RNA-sequencing analysis, we compared the effects of tamoxifen or DMSO control treatments on the transcriptomes of infected larvae at 2 dpi (3 days postfertilization [dpf]) and noninfected control larvae. Following quality control analysis and data processing (Fig. S2A to G), we analyzed the differential expression of transcripts in infected compared to noninfected larvae in the absence of tamoxifen. We found 204 genes to be differentially expressed during mycobacterial infection at 2 dpi (Supplemental Data S1 at https://doi.org/10.5281/zenodo.5788543), including upregulation of the matrix metalloproteinase genes *mmp13a* and *mmp9* (Fig. S3A), which is consistent with earlier transcriptomic data of *Mm*-infected zebrafish at the same time point after infection (47). Tamoxifen treatment of noninfected larvae caused differential expression of 141 genes, including genes involved in estrogen receptor (ER) signaling and autophagy and other cellular stress pathways, consistent with known effects of tamoxifen exposure (Supplemental Data S1 at https://doi.org/10.5281/zenodo.5788543) (24, 25, 31, 48–50).

10.1128/mbio.03024-22.3 FIG S2 Processing and quality control of zebrafish RNA-sequencing data. (A) Principal-component analysis (PCA) of transcriptomes of uninfected and *Mm*-infected zebrafish larvae treated with 5 μM tamoxifen or control (DMSO at equal volume). Treatment was started at 1 hpi, and isolation of RNA for RNA sequencing was performed at 2 dpi. Clustering of the different samples was driven by parent pairs over treatment or infection. Groups A to D indicate parent pairs, and each dot indicates a sample. Based on this result, we added the variable genotype to reflect that the major differences between samples are driven by parent pairs in our analysis. (B) *P* value histogram of the differential expression analysis of zebrafish larvae treated with 5 μM tamoxifen compared to treatment with control (DMSO at equal volume), as in A. Histogram shape, which before adjusting for false-discovery rates reveals test performance, showed a hill-shaped, as opposed to a uniform, distribution, implying that the data do not fit the test assumption. (C) Volcano plot of the differential expression analysis of zebrafish larvae treated with 5 μM tamoxifen compared to treatment with control (DMSO at equal volume), as in A. Red dots indicate significantly regulated genes (FDR-adjusted *P* value [padj] ≤ 0.05), while black dots indicate nonsignificantly regulated genes. The subset of genes in the wings of the plot have low read counts, high intersample variation, and high calculated fold changes. Further analysis determined these genes as artifacts. (D) Volcano plot of the differential expression analysis as in C using apeglm to reduce variance for genes with little information while preserving large differences. The more conservative analysis method apeglm shrinks the fold change to 0 for genes that contain insufficient information to accurately predict their fold change. The genes with a fold change of 0 form a vertical line. Red dots indicate significantly regulated genes (padj ≤ 0.05), while black dots indicate nonsignificantly regulated genes. (E) Volcano plot of the differential expression analysis as in D using *S* values. Red dots indicate significantly regulated genes (*S* value of ≤0.005), while black dots indicate nonsignificantly regulated genes. By using *S* values as opposed to FDR-adjusted *P* values, the wings as depicted in C or vertical line as depicted in D are no longer present. Based on their *S* values, these subsets of genes are deemed artifacts. (F) Correlation of transcriptomic data analyzed using FDR-corrected *P* values (as in D) compared to *S* values (as in E). Each dot indicates a gene, and the dashed lines indicate significance cutoffs. In the top left, there is a cluster of genes with low, significant adjusted *P* values but high, nonsignificant *S* values. (G) Zoom in of area boxed in F, indicating the genes that are significant by both *P* value (padj ≤ 0.05) and *S* value (*S* ≤ 0.005). Download FIG S2, TIF file, 1.8 MB. Copyright © 2022 Boland et al. 2022 Boland et al. https://creativecommons.org/licenses/by/4.0/ This content is distributed under the terms of the Creative Commons Attribution 4.0 International license.

10.1128/mbio.03024-22.4 FIG S3 Tamoxifen alters leukocyte-specific gene expression without affecting macrophage or neutrophil migration *in vivo.* (A and B) Interaction between tamoxifen treatment and infection in genes that are differentially regulated (*S* value of ≤0.005, as in Fig. S2E) and whose expression during infection was found to be dependent on tamoxifen treatment. Genes related to inflammation (A) and genes related to leukocyte function (B) are shown. Each dot represents the normalized gene read count of a single biological replicate (*n* = 10 larvae), while the line connects the means; uninf, uninfected. (C) Venn diagram showing the total number of genes differentially regulated by tamoxifen treatment in the absence of infection and by *Mm* infection in the absence of tamoxifen treatment. (D and E) Normalized gene read counts of genes whose expression was regulated (*S* ≤ 0.005) by both tamoxifen treatment and *Mm* infection individually. Genes related to immune function (D) and genes related to leukocyte function (E) are shown. Each dot represents the normalized gene read count of a single group of larvae (*n* = 10), while the line connects the means. (F) Leukocyte migration assay of mpeg1:mcherryF/mpx;GFP double transgenic zebrafish larvae treated with 5 μM tamoxifen or control (DMSO at equal volume). Treatment was started at 1 dpf. Larvae were anesthetized, and leukocyte migration was induced by tail amputation at 3 dpf. Representative stereo fluorescence images of leukocyte migration toward the injury (4 h postamputation) are shown. Cyan shows neutrophils (*mpx*:GFP), and magenta shows macrophages (*mpeg1*:mCherryF). The region of interest (ROI) indicates the area for quantification of leukocyte migration; scale bar, 220 μm. (G and H) Quantification of F, showing the number of migrated neutrophils (G) or macrophages (H). Each dot represents a single larva. Box plots with 95% confidence intervals are shown. The black line in the box plots indicates the group median, while the black line in the dot plot indicates the group mean. Statistical analysis was performed using a Mann-Whitney test. Download FIG S3, TIF file, 1.5 MB. Copyright © 2022 Boland et al. 2022 Boland et al. https://creativecommons.org/licenses/by/4.0/ This content is distributed under the terms of the Creative Commons Attribution 4.0 International license.

Next, we analyzed the genes that showed interaction between tamoxifen treatment and infection (i.e., genes whose expression during infection was altered by tamoxifen treatment) and found 28 significantly up- or downregulated genes (Table S2). These differential transcriptomic responses could be due to the lower bacterial burden in tamoxifen-treated larvae during infection than in the control group. For example, the lower upregulation of *mmp9* and *mmp13a* during tamoxifen treatment is in line with a reduced inflammatory response in larvae with lower infection burden (Fig. S3A). However, we also found alterations in leukocyte-specific marker genes that were dependent on tamoxifen treatment, such as *marco* and *mfap4*, suggesting that the number of leukocytes or their behavior during infection could be altered due to tamoxifen treatment (Fig. S3B). Furthermore, we found 14 genes that were differentially regulated (*S* value of ≤0.005) by both tamoxifen treatment and infection compared to their respective control larvae (Fig. S3C; Table S3). Interestingly, several of these 14 genes were related to immune processes (*cp* and *ccl34a*) (Fig. S3D) or were highly expressed in leukocytes (*mpx*, *grna*, and *mfap4*) (Fig. S3E). This indicated that tamoxifen treatment could modulate the cellular immune response even in the absence of infection. Together, these data correlate the decrease in bacterial burden in tamoxifen-treated larvae with modulation of inflammatory responses and leukocyte development or behavior.

10.1128/mbio.03024-22.7 TABLE S2 Interaction of treatment and infection on gene regulation. Download Table S2, PDF file, 0.1 MB. Copyright © 2022 Boland et al. 2022 Boland et al. https://creativecommons.org/licenses/by/4.0/ This content is distributed under the terms of the Creative Commons Attribution 4.0 International license.

10.1128/mbio.03024-22.8 TABLE S3 Effect of treatment and infection on gene regulation. Download Table S3, PDF file, 0.08 MB. Copyright © 2022 Boland et al. 2022 Boland et al. https://creativecommons.org/licenses/by/4.0/ This content is distributed under the terms of the Creative Commons Attribution 4.0 International license.

The development of *Mm* infection in zebrafish larvae depends strongly on migratory responses of macrophages and neutrophils, which aggregate to form the initial stages of TB granulomas (51–53). In addition to transcriptional effects on leukocyte markers detected in our study, tamoxifen has been reported to both inhibit and stimulate neutrophil migration (54–56). Therefore, we asked if leukocyte migration was altered after tamoxifen treatment in our model. To this end, we used an established injury-based migration assay, the tail amputation assay (57), in a double transgenic neutrophil and macrophage marker line and assessed the number of neutrophils and macrophages that migrated to the wound-induced site of inflammation. We did not find a significant difference between control and tamoxifen-treated groups in both neutrophil and macrophage numbers that migrated toward the injury (Fig. S3F to H). In conclusion, despite transcriptional changes in leukocyte-specific genes caused by tamoxifen treatment, we did not detect altered leukocyte behavior in response to an inflammatory stimulus. Therefore, we decided to focus next on analyzing the broad systemic effects of tamoxifen treatment detected in the RNA-sequencing analysis, specifically in relation to ER signaling and autophagy, processes both known to be modulated by tamoxifen.

### The host-directed effect of tamoxifen is independent of ER signaling.

**FIG 3:**
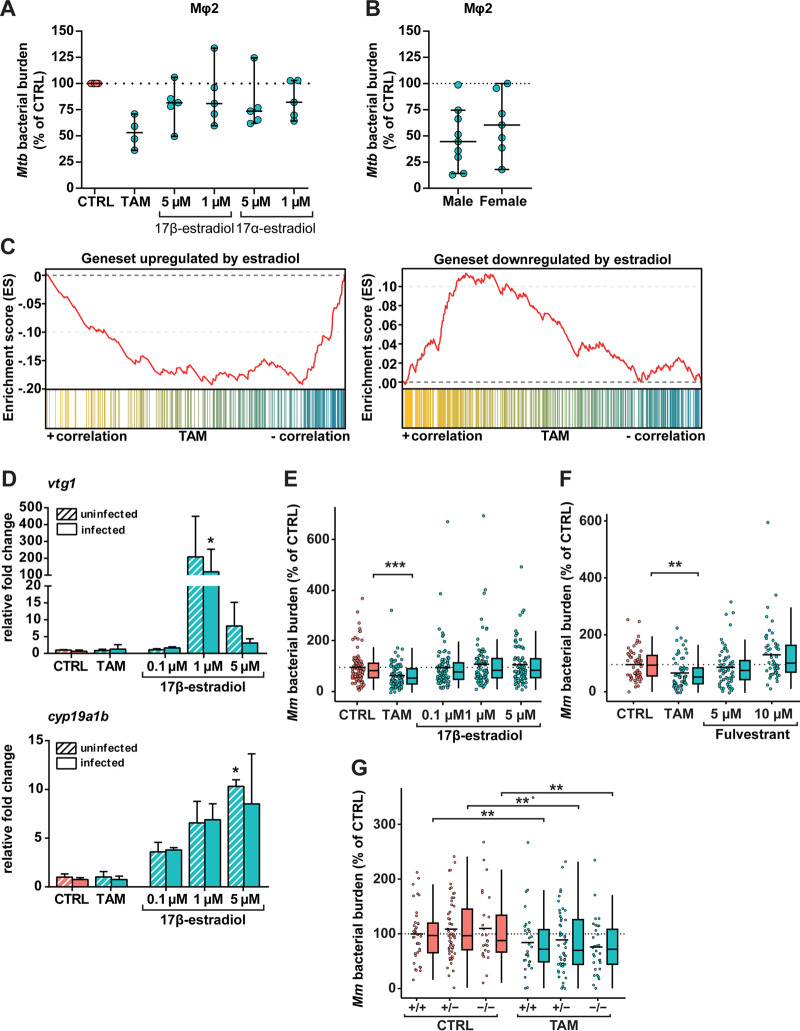
The host-directed effect of tamoxifen is independent of ER signaling. (A) CFU assay of Mφ2 macrophages infected with H37Rv-*Mtb* and treated with tamoxifen (10 μM), 17β-estradiol (1 or 5 μM), 17α-estradiol (1 or 5 μM), or control (DMSO at equal volume) for 24 h. Each dot represents a single donor (4 to 5 donors were tested) and depicts the mean of 3 replicates normalized to control. The dotted line indicates the control set at 100%, and median values + 95% confidence intervals are shown for every condition. Statistical significance was tested using a Wilcoxon matched-pairs signed-rank test with *post hoc* Benjamini-Hochberg correction. (B) CFU assay of Mφ2 macrophages infected with H37Rv-*Mtb* and treated with 10 μM tamoxifen for 24 h separated for donor sex. The graph includes data points from A and Fig. 1A. Each dot represents a single donor (9 in the male group and 7 in the female group) and depicts the mean of 3 replicates normalized to control. The dotted line indicates the control set at 100%, and median values + 95% confidence intervals are shown for every condition. Statistical significance was tested using a Mann-Whitney *U* test. (C) GSEA enrichment plots of downregulated (left) and upregulated (right) estradiol-responding genes in zebrafish larvae treated with 5 μM tamoxifen for 2 days (3 dpf). All estradiol-responding genes in the tamoxifen-treated larval transcriptome were ranked according to their statistical significance and direction of regulation from left (most significant, upregulated in yellow) to right (most significant, downregulated in blue). Each column depicts the position of an individual gene belonging to the gene set of estradiol-responding genes in the ranked list. (D) Noninfected and mWasabi-expressing *Mm*-infected zebrafish larvae were treated with 5 μM tamoxifen, increasing doses of the ER agonist 17β-estradiol (0.1, 1, and 5 μM), or control (DMSO at 0.05% [vol/vol]) starting at 1 hpi. Transcript levels of two β-estradiol-responsive genes, *vtg1* (top) and *cyp19a1b* (bottom) were determined by qPCR analysis at 4 dpi. Data were normalized to the expression of the housekeeping gene TATA box binding protein (*tbp*), and data of 3 biological replicates were combined (*n* = 10 larvae per replicate). Each bar depicts the average fold change (FC) of transcript levels relative to noninfected or infected control-treated zebrafish larvae, and the error bar indicates standard error of the mean (SEM). Statistical analysis was performed using a Kruskal-Wallis test with Dunn’s multiple-comparison test. The effect of treatment compared to control was analyzed within the noninfected and infected groups separately. (E) Bacterial burden assay of mWasabi-expressing *Mm*-infected zebrafish larvae treated as in D. Treatment was started at 1 hpi, and larvae were anesthetized at 4 dpi for imaging. Bacterial burden was normalized to the control, and data of 3 experimental repeats were combined (*n* = 93 to 95 per group). Each dot represents a single larva. Box plots with 95% confidence intervals are shown. The black line in the box plots indicates the group median, while the black line in the dot plot indicates the group mean. The dotted line indicates control mean set at 100%. Statistical analysis was performed using a Kruskal-Wallis test with Dunn’s multiple-comparison test. (F) Bacterial burden assay of mWasabi-expressing *Mm*-infected zebrafish larvae treated with of 5 μM tamoxifen, increasing doses of the ER antagonist fulvestrant (5 and 10 μM), or control (DMSO at 0.05% [vol/vol]). Treatment was started at 1 hpi, and larvae were anesthetized at 4 dpi for imaging. Bacterial burden was normalized to the control, and data from 2 experimental repeats were combined (*n* = 53 to 63 per group). Each dot represents a single larva. Box plots with 95% confidence intervals are shown. The black line in the box plots indicates the group median, while the black line in the dot plot indicates the group mean. The dotted line indicates the control mean set at 100%. Statistical analysis was performed using a Kruskal-Wallis test with Dunn’s multiple-comparison test. (G) Bacterial burden assay of mWasabi-expressing *Mm*-infected *esr2_b_^+/+^*, *esr2_b_^+/−^*, and *esr2b*^−/−^ zebrafish larvae treated with 5 μM tamoxifen or control (DMSO at equal volume). Treatment was started at 1 hpi, and larvae were anesthetized at 4 dpi for imaging. Bacterial burden was normalized to the control, and data from 2 experimental repeats were combined (*n* = 25 to 55 per group). Each dot represents a single larva. Box plots with 95% confidence intervals are shown. The black line in the box plots indicates the group median, while the black line in the dot plot indicates the group mean. The dotted line indicates the mean of control-treated *esr2_b_^+/+^* zebrafish larvae set at 100%. Statistical significance of the difference between the control and tamoxifen-treated groups was determined using a two-way ANOVA; *, *P* < 0.05; **, *P* < 0.01; ***, *P* < 0.001.

The ER is the most well-characterized target of tamoxifen. Tamoxifen is known to have a dual role and can act both as an agonist and as an antagonist of ER signaling (24). We therefore investigated whether modulating ER signaling by selective ER-agonistic and ER-antagonistic compounds would affect bacterial burden and in which direction. Having shown that tamoxifen reduced bacterial burden in MelJuSo cells, primary Mφ1 and Mφ2 macrophages, and *in vivo*, we decided to focus our mechanistic studies in one of the primary macrophage cell types while using the *in vivo* model for complementary analyses. For this purpose, we selected Mφ2 macrophages because these are easier to infect than Mφ1macrophages and better resemble the alveolar macrophages that are the target of *Mtb* during clinical infection (45, 46). Human Mφ2 macrophages were infected with *Mtb* and treated with the ER agonists 17α-estradiol or 17β-estradiol, and bacterial outgrowth was measured by CFU assay. While tamoxifen reduced *Mtb* growth by approximately 50% compared to control DMSO, neither ER agonist consistently affected *Mtb* growth, regardless of the concentration used (Fig. 3A). Because tamoxifen effects may depend on sex differences (58), and macrophage ER receptor levels differ between sexes (59), we investigated whether human donor sex influenced tamoxifen HDT activity in primary macrophages from male versus female donors. No significant differences in tamoxifen’s efficacy against intracellular bacteria were observed between male and female primary macrophages (Fig. 3B).

Next, we studied if ER signaling is responsible for the effect of tamoxifen on *Mm* infection *in vivo*. We used our zebrafish transcriptome data to identify classes of differentially expressed genes associated with tamoxifen treatment by performing gene set enrichment analysis (GSEA) (60). Tamoxifen-treated larvae showed downregulation of genes normally upregulated by 17β-estradiol, while genes that are normally downregulated by this ER agonist were upregulated after tamoxifen treatment (Fig. 3C). Considering this large effect of tamoxifen on ER target genes, we investigated whether activating or blocking ER signaling *in vivo* would result in a similar reduction of bacterial burden as tamoxifen treatment. We first used 17β-estradiol to activate ER signaling during *Mm* infection. To verify the effect of this agonist, we analyzed the expression level of two ER target genes (vitellogenin 1 [*vtg1*] and cytochrome P450, family 19, subfamily A, polypeptide 1 [*cyp19a1b*]), by reverse transcription-quantitative PCR (qPCR). After 17β-estradiol treatment (at 0.1, 1, and 5 μM), both infected and noninfected larvae showed markedly increased expression levels of *cyp19a1b* (reaching approximately 10-fold at the highest 17β-estradiol dose) and *vtg1* (approximately 100-fold at the 1 μM dose), while upregulation of these genes was not detected following treatment with tamoxifen compared to the control treatment (Fig. 3D). Despite activation of ER signaling, no reduction in bacterial burden could be observed following 17β-estradiol treatment, while treatment with tamoxifen reduced bacterial burden significantly compared to the control (Fig. 3E). These results led us to consider the possibility that the effect on bacterial burden after tamoxifen treatment might be due to the ER-antagonistic role of tamoxifen. Therefore, we investigated whether we could reproduce the effect of tamoxifen using the ER antagonist fulvestrant. However, we did not detect an effect on bacterial burden using two different doses of fulvestrant (Fig. 3F). In contrast, we even observed a trend toward an increase of bacterial burden with the higher dose of fulvestrant (10 μM). These data indicate that even though tamoxifen treatment alters the host transcriptome related to ER signaling, activating or blocking ER signaling does not enhance the host response to mycobacterial infection, suggesting that tamoxifen controls bacterial burden via alternative mechanisms than ER signaling.

To provide further evidence that tamoxifen indeed functions independently of ER signaling, we followed a genetic approach. Two ER subtypes, ERα and ERβ, are conserved in vertebrate evolution. In zebrafish, *esr1* encodes the ERα subtype, while, due to a gene duplication event, two ER genes (*esr2a* and *esr2b*) encode ERβ (61, 62). For our study, we took advantage of an available *esr2b* loss-of-function mutant, which previously has been shown to be impaired in its response to viral infection (63). We observed reduced *Mm* bacterial burdens in all tamoxifen-treated groups compared to the DMSO control-treated groups independently of the *esr2_b_^+/+^*, *esr2_b_^+/−^*, and *esr2b*^−/−^ genotypes (Fig. 3G). Together, the pharmacological and genetic data show that neither activating nor blocking ER signaling in zebrafish leads to a reduction of bacterial burden and that Esr2b is not required for the effect of tamoxifen on bacterial burden.

Collectively, these data suggest that tamoxifen’s HDT effect against intracellular bacteria is likely independent of ER signaling. In line with this result, ER agonists do not consistently affect intracellular *Mtb* growth, the activity of the ER pathway in zebrafish is not required for tamoxifen’s HDT effect, and its efficacy in primary macrophages is not affected by the sex of the donor.

### Tamoxifen treatment modulates autophagy in infected human macrophages and zebrafish.

**FIG 4:**
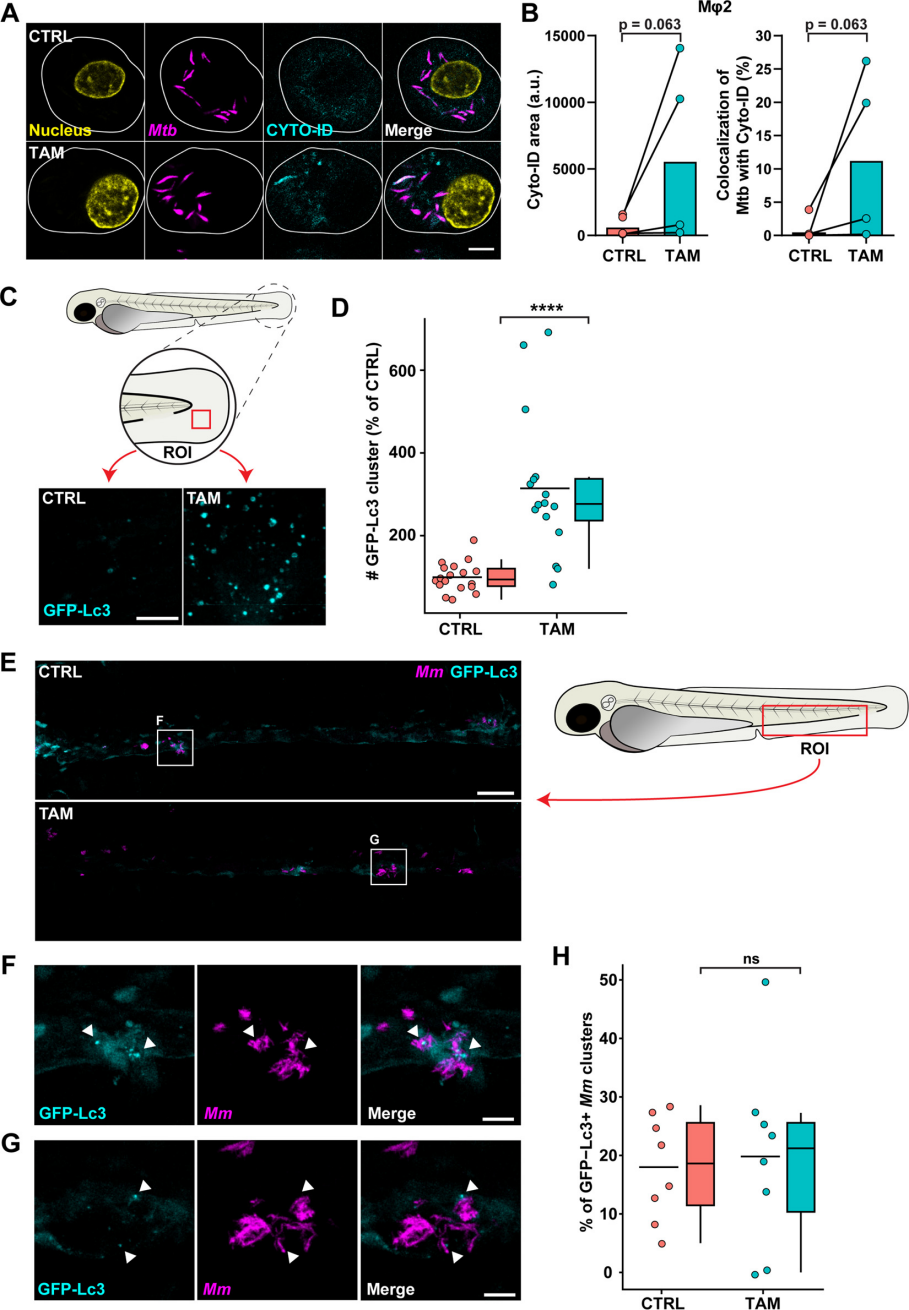
Tamoxifen treatment modulates autophagy in infected human macrophages and zebrafish. (A) Confocal microscopy of DsRed-expressing H37Rv-*Mtb*-infected Mφ2 macrophages treated with 10 μM tamoxifen or control (DMSO at equal volume) for 4 h. Thirty minutes before the experimental endpoint, cells were incubated with Cyto-ID to stain for autophagy-related vesicles, fixed with 1% paraformaldehyde, and counterstained for the nucleus using Hoechst 33342. In the representative images, yellow shows the nucleus, magenta shows *Mtb*, and cyan shows autophagy-related vesicles; scale bar, 5 μm. (B) Quantification of Cyto-ID signals in A. Cyto-ID-positive area (left) and *Mtb* colocalization with Cyto-ID-positive vesicles (right) are displayed. Each dot displays the mean of 3 or 4 replicates and represents a single donor (4 donors in total), with the median indicated by colored bars. Statistical significance was tested using a Wilcoxon matched-pairs signed-rank test; a.u., arbitrary units. (C) Confocal microscopy of transgenic GFP-Lc3 zebrafish larvae treated with 5 μM tamoxifen or control (DMSO at equal volume). Treatment was started at 3 dpf, and larvae were fixed with 4% paraformaldehyde at 4 dpf for imaging. Representative max projection images of GFP-Lc3-positive vesicles in the indicated region of imaging (ROI) in the tail fin are shown. Cyan shows GFP-Lc3-positive vesicles; scale bar, 10 μm. (D) Quantification of GFP-Lc3 structures shown in C. Data were normalized to the control, and data from 2 experimental repeats were combined (*n* = 16 to 18 per group). Each dot represents a single larva. Box plots with 95% confidence intervals are shown. The black line in the box plots indicates the group median, while the black line in the dot plot indicates the group mean. Statistical analysis was performed using a Mann-Whitney test. (E) Confocal microscopy of mCherry-expressing *Mm*-infected transgenic GFP-Lc3 zebrafish larvae treated with 5 μM tamoxifen or control (DMSO at equal volume). Treatment was started at 1 hpi, and at 2 dpi, larvae were fixed with 4% paraformaldehyde for imaging. Representative max projection images of the ROI in the caudal hematopoietic tissue (CHT) region are shown. Cyan shows GFP-Lc3-positive vesicles, and magenta shows *Mm*; scale bar, 50 μm. (F and G) Enlargement of areas indicated in E. Cyan shows GFP-Lc3-positive vesicles, and magenta shows *Mm*. Arrowheads indicate GFP-Lc3-positive *Mm* clusters; scale bar, 10 μm. (H) Quantification of GFP-Lc3-positive *Mm* clusters in the CHT region shown in E normalized to the control (*n* = 8 per group). Each dot represents a single larva. Box plots with 95% confidence intervals are shown. The black line in the box plots indicates the group median, while the black line in the dot plot indicates the group mean. Statistical analysis was performed using a Mann-Whitney test; ****, *P* < 0.0001; ns, not significant.

Because tamoxifen induces and modulates autophagy and because autophagy contributes to host defense against TB, we next investigated the role of tamoxifen-induced autophagy in inhibiting bacterial outgrowth (31, 50, 64). We first used Cyto-ID, a tracer for autophagy-related vesicles offering the advantage of staining all intracellular autophagy-related vesicles independent of proteins such as LC3. Mφ2 macrophages were infected with *Mtb*, treated for 4 h with tamoxifen, stained with the Cyto-ID tracer, and visualized using confocal microscopy (Fig. 4A). Although differences did not reach the statistical significance threshold due to well-known variation between human donors, clearly increasing trends in area of Cyto-ID vesicles and colocalization of *Mtb* with these vesicles were observed in response to tamoxifen (Fig. 4B).

We further explored the role of autophagy using a fluorescent zebrafish reporter line for Lc3 (65). Zebrafish larvae (3 dpf) were treated with tamoxifen for 24 h, and the enhanced green fluorescent protein (EGFP)-map1lc3b (GFP-Lc3) response was visualized in the thin tissue of the larval tail fin, which is well suited for using confocal microscopy (Fig. 4C) (42). We observed a significant increase in GFP-Lc3-positive structures in the tamoxifen-treated group compared to in the control-treated group (Fig. 4D), consistent with an autophagy-modulating effect of tamoxifen. In contrast, neither the ER agonist 17β-estradiol nor the ER antagonist fulvestrant showed an increase in GFP-Lc3-positive structures (Fig. S4A and B). Therefore, we conclude that tamoxifen modulates autophagy in the zebrafish model by an ER-independent mechanism. Next, to study whether tamoxifen increased colocalization between GFP-Lc3 structures and *Mm*, we infected 1 dpf embryos of the GFP-Lc3 reporter line and imaged them at 2 dpi in their caudal hematopoietic tissue (CHT), a preferred location for aggregation of infected macrophages, the initial step in tuberculous granuloma formation (51). We observed *Mm* clusters distributed from the injection site to the end of the tail and various GFP-Lc3-positive structures colocalized with these clusters (Fig. 4E to G). However, we found no significant differences in the percentage of *Mm* clusters positive for GFP-Lc3 structures between control and tamoxifen treatments (Fig. 4H).

10.1128/mbio.03024-22.5 FIG S4 Autophagy is not modulated by ER ligands in zebrafish. (A) Confocal microscopy of transgenic GFP-Lc3 zebrafish larvae treated with 5 μM tamoxifen, 5 μM 17β-estradiol (ER agonist), 5 μM fulvestrant (ER antagonist), or control (DMSO at equal volume). Treatment was started at 3 dpf, and larvae were fixed with 4% paraformaldehyde at 4 dpf. Representative images of GFP-Lc3-positive vesicles in the tail fin are shown. Cyan shows GFP-Lc3-positive vesicles; scale bar, 10 μm. (B) Quantification of GFP-Lc3 signal in A. Data were normalized to the control, and data from 2 experimental repeats were combined (*n* = 14 to 16 per group). Each dot represents a single larva. Box plots with 95% confidence intervals are shown. The black line in the box plots indicates the group median, while the black line in the dot plot indicates the group mean. The dotted line indicates the control mean. Statistical analysis was performed using a Kruskal-Wallis test with Dunn’s multiple-comparison test; **, *P* < 0.01. Download FIG S4, TIF file, 1.4 MB. Copyright © 2022 Boland et al. 2022 Boland et al. https://creativecommons.org/licenses/by/4.0/ This content is distributed under the terms of the Creative Commons Attribution 4.0 International license.

In summary, tamoxifen treatment increased the abundance of autophagy vesicles both *in vitro* and *in vivo*, but the effects on colocalization of these vesicles with mycobacteria were modest or undetectable. This suggests that autophagy induction by tamoxifen might play a secondary or temporary role in decreasing mycobacterial infection.

### Tamoxifen treatment alters lysosomal function and increases mycobacterial lysosomal localization *in vitro* and *in vivo*.

**FIG 5:**
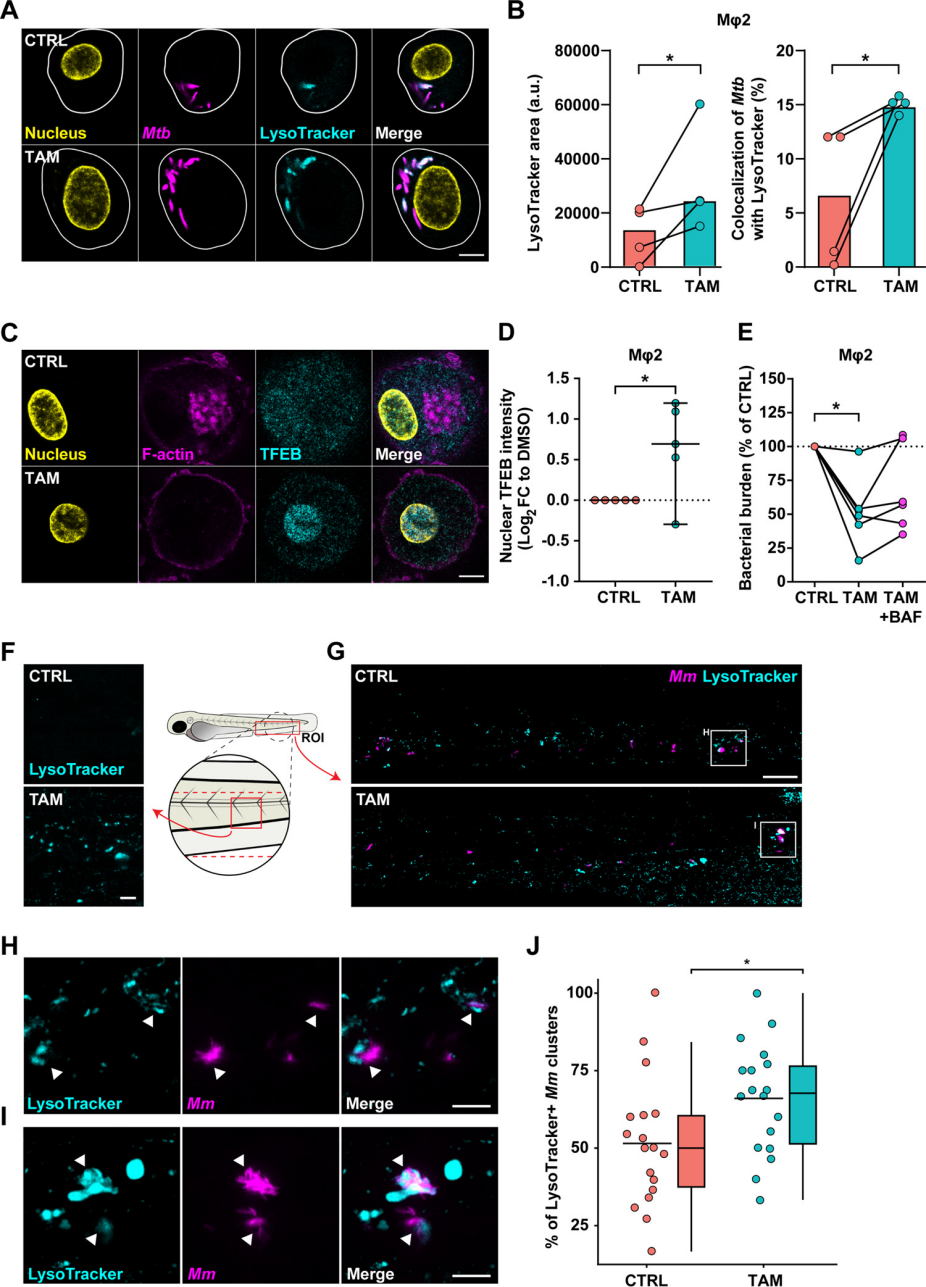
Tamoxifen treatment alters lysosomal function and increases mycobacterial lysosomal localization *in vitro* and *in vivo*. (A) Confocal microscopy of DsRed-expressing H37Rv-*Mtb*-infected Mφ2 macrophages treated with 10 μM tamoxifen or DMSO at equal volume for 4 h. Thirty minutes before the experimental endpoint, cells were incubated with LysoTracker Deep Red to stain for acidic vesicles, fixed with 1% paraformaldehyde, and counterstained for the nucleus using Hoechst 33342. In the representative images, yellow shows the nucleus, magenta shows *Mtb*, and cyan shows acidic vesicles; scale bar, 5 μm. (B) Quantification of LysoTracker signal in A. Lysotracker-positive area (left) and *Mtb* colocalization with Lysotracker-positive vesicles (right) are shown. Each dot displays the mean of 3 or 4 replicates and represents a single donor (4 donors in total), with the median indicated by colored bars. Statistical significance was tested using a paired *t* test; a.u., arbitrary units. (C) Confocal microscopy of DsRed-expressing *Mtb*-infected Mφ2 macrophages treated with 10 μM tamoxifen or DMSO at equal volume for 4 h. Cells were fixed at the experimental endpoint, permeabilized using 0.1% Triton-X, stained for TFEB, and counterstained for the nucleus and F-actin using Hoechst 33342 and phalloidin, respectively; scale bar, 5 mm. (D) Quantification of nuclear TFEB intensity in C. Each dot displays the log_2_ (FC) of median nuclear TFEB intensity per donor normalized to DMSO (5 donors in total); 95% confidence intervals are indicated. Statistical significance was tested using a paired *t* test. (E) CFU assay of Mφ2 macrophages infected with H37Rv-*Mtb* and treated for 24 h with 10 μM tamoxifen, 10 μM tamoxifen in combination with 10 nM bafilomycin (BAF), or control (DMSO at equal volume). Each dot represents a single donor (6 donors in total) and depicts the mean of 3 replicates. Statistical significance was tested using an RM one-way ANOVA with Holm-Sidak multiple-testing correction. (F) Confocal microscopy maximum projection of the indicated ROI in zebrafish larvae treated with 5 μM tamoxifen or control (DMSO at equal volume). Treatment was started at 31 hpf, and at 3 dpf, larvae were immersed in 5 μM LysoTracker Red DND-99 for 1 h and subsequently anesthetized for imaging. Cyan shows acidic vesicles; scale bar, 10 μm. (G) Confocal microscopy maximum projection of mWasabi-expressing *Mm*-infected zebrafish larvae treated with 5 μM tamoxifen or control (DMSO at equal volume). Treatment was started at 1 hpi, and at 2 dpi, larvae were immersed in 5 μM LysoTracker Red DND-99 for 1 h and subsequently anesthetized for imaging. Representative maximum projection images of LysoTracker-positive *Mm* clusters in the CHT region are shown. Cyan shows acidic vesicles, and magenta shows *Mm*; scale bar, 50 μm. (H and I) Enlargement of areas indicated in G. Cyan shows acidic vesicles, and magenta shows *Mm*. Arrowheads indicate LysoTracker-positive *Mm* clusters; scale bar, 10 μm. (J) Quantification of LysoTracker-positive *Mm* clusters normalized to the control. Data from 3 experimental repeats were combined (*n* = 18 per group). Each dot represents a single larva. Box plots with 95% confidence intervals are shown. The black line in the box plots indicates the group median, while the black line in the dot plot indicates the group mean. Statistical analysis was performed using a Mann-Whitney test; *, *P* < 0.05.

Because mycobacteria can be targeted to lysosomes both dependent and independent of autophagy, we investigated whether vesicle maturation was affected by tamoxifen. Therefore, a tracer for lysosomes, LysoTracker, was used to quantify acidic vesicles. Mφ2 macrophages were infected with *Mtb*, treated for 4 h with tamoxifen, stained with LysoTracker, and visualized using confocal microscopy (Fig. 5A). Tamoxifen consistently and significantly increased both LysoTracker area and the colocalization of *Mtb* with LysoTracker (Fig. 5B), suggesting that tamoxifen’s effect on the lysosomal response is relevant for *Mtb* infection. To confirm that tamoxifen increased lysosomal activity, nuclear accumulation of transcription factor EB (TFEB), a master regulator of the coordinated lysosomal expression and regulation (CLEAR) gene network and autophagy (66, 67), was visualized using confocal microscopy after 4 h of treatment (Fig. 5C). Tamoxifen significantly increased nuclear accumulation of TFEB (Fig. 5D).

To investigate whether tamoxifen’s induction of the lysosomal response is (partly) responsible for decreased bacterial survival, the lysosomal activity inhibitor bafilomycin was used. Mφ2 macrophages were infected with *Mtb* and treated for 24 h with tamoxifen or tamoxifen supplemented with 10 nM bafilomycin (Fig. 5E). Tamoxifen treatment showed a decrease of *Mtb* outgrowth as expected, while this decrease was instead consistently impacted (*P* = 0.16) by the addition of bafilomycin, implicating the lysosomal response in tamoxifen’s mechanism of action.

In line with these results, further analysis of our zebrafish transcriptome data by means of pathway enrichment against the KEGG database revealed phagosome and lysosome pathways as strongly enriched in response to tamoxifen treatment (Table S4). In addition, Gene Ontology (GO) and gene set enrichment analysis (GSEA) showed that genes with molecular functions such as hydrolase and peptidase activity, biological processes such as proteolysis, and genes belonging to the lysosome compartment were enriched in response to tamoxifen treatment (Table S4). In fact, lysosomal genes, such as those encoding V-ATPases and cathepsins and *lamp1*, were all upregulated in the transcriptomes of tamoxifen-treated larvae (Supplemental Data S1 at https://doi.org/10.5281/zenodo.5788543). We therefore asked whether tamoxifen treatment could also increase the localization of *Mm* clusters in lysosomes *in vivo*. Thus, we treated embryos with tamoxifen or DMSO starting at 1 dpf (1 h postinfection [hpi]) for 2 days and performed LysoTracker staining at 2 dpi (3 dpf). A strong increase in LysoTracker signal intensity was observed independent of infection (Fig. 5F). Furthermore, imaging of the CHT region of infected larvae (Fig. 5G to I) showed an increase in the colocalization between LysoTracker signal and *Mm* clusters from 50% in the control group to 65% in tamoxifen-treated larvae (Fig. 5J), corroborating the transcriptomic results that tamoxifen modulates lysosomal activity. While results in *Mtb*-infected macrophages had shown that inhibiting lysosomal function interfered with the effect of tamoxifen (Fig. 5E), toxicity of the bafilomycin-tamoxifen combination to zebrafish embryos prevented us from confirming this *in vivo.*

10.1128/mbio.03024-22.9 TABLE S4 KEGG pathway and Gene Ontology (GoSeq) analysis. Download Table S4, PDF file, 0.10 MB. Copyright © 2022 Boland et al. 2022 Boland et al. https://creativecommons.org/licenses/by/4.0/ This content is distributed under the terms of the Creative Commons Attribution 4.0 International license.

Taken together, both in primary human macrophages and in zebrafish, we observed increased LysoTracker signal intensity following treatment with tamoxifen. Tamoxifen led to enhanced targeting of *Mtb* and *Mm* to LysoTracker-positive vesicles in these *in vitro* and *in vivo* infection models. Additionally, this effect of tamoxifen treatment was associated with modulated lysosomal gene expression. Importantly, using the *in vitro* model, we showed that bafilomycin cotreatment inhibited tamoxifen’s efficacy on bacterial survival. These data lead us to propose that tamoxifen treatment reduces infection burden during mycobacterial infection by a host-dependent increase of lysosomal activity.

## DISCUSSION

The concept of HDT, combating infection with drugs that empower the immune system, is increasingly explored as alternative or adjunctive therapeutic approaches against *Mtb* strains that are unresponsive to classical antibiotics (13–15, 22). We demonstrate that the breast cancer therapy drug tamoxifen restricts bacterial outgrowth of *Mtb* in primary human macrophages of different inflammatory states and differentiation stages. Furthermore, tamoxifen shows high efficacy against clinical isolates of MDR-TB and is also active against *Stm*. Importantly, using *Mm*-infected zebrafish larvae as an *in vivo* TB model, we further substantiated the repurposing potential of tamoxifen as an HDT for TB. Furthermore, we showed that tamoxifen reduces bacterial burden independent of ER signaling and propose that the HDT effect of tamoxifen is mediated primarily by enhancing lysosomal degradation pathways.

Tamoxifen has been proposed as a new antibiotic because it was found to have direct antibacterial effects against intracellular pathogens (27–29). Tamoxifen antimicrobial activity against *Mtb* was found at doses ranging from 16.7 to 26.8 μM, whereas lower doses similar to the ones used in our study lacked significant effects (29, 30). In agreement with this, when using tamoxifen in low doses up to 10 μM, we only observed direct effects on either *Mtb* or *Mm* growth in liquid cultures after incubation times longer than the treatment times used in the experimental settings, while, importantly, both *Mtb* and *Stm* outgrowth and *Mm* burden in infected human macrophages and zebrafish larvae, respectively, were inhibited effectively by tamoxifen at these doses (5 to 10 μM). This is additionally strengthened by the finding that inhibition of host lysosomal activity decreases the efficacy of tamoxifen in *Mtb*-infected macrophages. Therefore, we propose tamoxifen as a potential new HDT against (myco)bacterial infection. Recently, a structurally and functionally related breast cancer drug bazedoxifene has also been proposed as an HDT for TB, supporting the therapeutic potential of this class of chemicals (68). The therapeutic potential of these drugs may extend to a wide range of bacterial pathogens, as tamoxifen was recently found to have an immunomodulatory effect against MDR Gram-negative bacilli, including Acinetobacter baumannii, Pseudomonas aeruginosa, and Escherichia coli (69).

The best-known target of drugs like tamoxifen and bazedoxifene is the ER (24, 25). However, our data do not support a role for the ER in mediating the antimycobacterial function of tamoxifen. ER agonists did not significantly affect bacterial outgrowth *in vitro*, and donor sex did not affect tamoxifen-restricted *Mtb* outgrowth in our model, despite that sex-based differences both in macrophage ER receptor amounts and differential effects of tamoxifen treatment have been reported (58, 59). Furthermore, although zebrafish transcriptomic analysis showed that tamoxifen antagonized ER signaling, tamoxifen reduced *Mm* burden in *esr2b* mutants, indicating that its HDT effect is independent of the Esr2b receptor. Although we cannot exclude the involvement of other zebrafish ER receptors (*esr2a* and *esr1*), we considered the Esr2b receptor a prime candidate for mediating a potential HDT effect of tamoxifen because an *esr2b* loss-of-function mutant has previously been linked to a host defense phenotype (63). Finally, chemical activation and inhibition of ER signaling using 17β-estradiol and fulvestrant, respectively, did not affect mycobacterial burden, while they are known to affect ER signaling in zebrafish (70, 71). Of note, we show several other host pathways, including autophagy and lysosome function, to be modulated by tamoxifen in addition to ER signaling. Thus, we propose that ER-independent host-directed effects of tamoxifen are responsible for the reduction of bacterial burden. It will require further investigation to understand how these ER-independent pathways might be implicated in tamoxifen-based cancer therapy or may affect the outcome of experiments using tamoxifen-inducible gene expression systems.

The autophagy-inducing function of tamoxifen, demonstrated in several previous studies (31, 50), could be a plausible explanation for its antimycobacterial effect, considering that activating autophagy reduces mycobacterial burden both *in vitro* and *in vivo* (20, 41). Furthermore, *Mtb* expresses several autophagy-inhibiting virulence factors, which is a clear indication that this pathogen must defend itself against autophagy (72). However, the interaction between mycobacteria and autophagy is not well understood, and, therefore, the potential therapeutic effects that may be expected from autophagy-inducing therapies remain unclear (19). While several studies found impaired autophagy during mycobacterial infection to be detrimental to the host, it has also been found that mutation of a number of autophagy-related genes did not affect the outcome of infection in a mouse TB model (43, 73–75). Of note, the anti-TB effect of the related drug bazedoxifene was attributed to its autophagy-inducing properties, dependent on AKT/mTOR signaling and the production of mitochondrial reactive oxygen species (ROS) (68). The authors proposed that bazedoxifene suppresses *Mtb* outgrowth in macrophages by enhancing autophagosome formation, as chemical or genetic inhibition of autophagy reduced the antibacterial effect. Likewise, we also observed autophagy-increasing effects of tamoxifen both *in vitro* and *in vivo*, which might similarly be related to mitochondrial ROS production, as our transcriptomic data indicated a mitochondrial stress response, which is a well-known effect of tamoxifen (48, 49). However, while our results suggested increased *Mtb* localization in Cyto-ID-positive vesicles *in vitro*, we were unable to demonstrate an increase in the colocalization of *Mm* with the autophagy marker GFP-Lc3 *in vivo*. This might be explained by both the transient nature of GFP-Lc3-*Mm* associations and the properties of the tracer Cyto-ID, which stains not only Lc3-positive vesicles but also a broader range of autophagy-related vesicles as well as autophagolysosomes (76), thus possibly reflecting mature lysosomes with degraded Lc3. Taken together, it remains possible that increased autophagosome formation contributes to the HDT effect of tamoxifen, but, based on our data on autophagy and on the lysosomal responses, we favor the hypothesis that tamoxifen restricts mycobacterial growth primarily by augmenting the lysosomal defense.

The clearance of (auto)phagosomes as well as the delivery of neoantimicrobial peptides depends on the fusion with lysosomes, resulting in autophagolysosomes or phagolysomes (16, 77, 78). Even in the absence of infection, the transcriptome of tamoxifen-treated zebrafish showed major modulation of lysosomal function. Although studies in breast cancer cells have shown that tamoxifen inhibits lysosomal acidification early during treatment due to its lysosomotrophic behavior (79–81), importantly, this rapidly triggers lysosomal activation that within hours restores pH and increases lysosomal volume to a level higher than before treatment (81). In agreement, LysoTracker signal increased after tamoxifen treatment in both human macrophages and zebrafish larvae. This appears to have a positive effect on infection control, as we observed increased colocalization between LysoTracker signal and both *Mtb in vitro* and *Mm* clusters *in vivo*. Additionally, we showed that *in vitro*, 4 h of tamoxifen treatment induced nuclear accumulation of TFEB, which is a master transcriptional regulator of the lysosomal response, but interestingly also induced autophagosome formation (82). Finally, the lysosomal activity inhibitor bafilomycin was shown to partly abrogate the effect of tamoxifen on bacterial burden. Based on these results, we hypothesize that in tamoxifen-treated cells, mycobacteria are sequestrated more easily to lysosomes due to their higher abundance or activity or that the mycobacteria are less likely to escape the lysosome or arrest lysosomal maturation. Further work could investigate the role of other potential mechanisms involved, such as the ones described in the recently published exhaustive review of tamoxifen’s ER-independent effects in macrophages and tamoxifen’s mechanism of action in the treatment of leishmaniasis (83, 84).

Taken together, our results support that the increase in lysosomal activation by the lysosomotrophic drug tamoxifen empowers the host to better control intracellular infection with various intracellular pathogens, including *Mtb* and *Stm*, and that this underlies the host-mediated therapeutic effect observed in mycobacterial *in vitro* and *in vivo* infection models. This therapeutic effect, which enhances the killing capacity of macrophages, may be augmented by other immunomodulatory functions of tamoxifen, which were recently described, including the reduction of inflammatory cytokine release and the stimulation of neutrophil extracellular trap formation (54, 69). Treatment with tamoxifen vastly reduced *Mtb* outgrowth in primary human macrophages, while in combination with a low dose of rifampicin affected *Mtb* outgrowth with close to a 2-log reduction. Importantly, 4 days of tamoxifen monotherapy in the zebrafish model for TB achieved an average reduction of the overall infection load by 30%. Tamoxifen is therefore a prime candidate for further evaluation as an adjunctive therapy to classical antibiotics, particularly for MDR- and XDR-TB.

## MATERIALS AND METHODS

### Chemicals and antibodies.

Tamoxifen citrate (tamoxifen) and rifampicin were purchased from Sigma-Aldrich (Zwijndrecht, The Netherlands). Isoniazid was purchased from SelleckChem (Munich, Germany). Gentamicin sulfate was bought from Lonza BioWhittaker (Basel, Switzerland), and hygromycin B was acquired from Life Technologies-Invitrogen (Bleiswijk, The Netherlands). Fulvestrant, 17β-estradiol, and Hoechst 33342 were purchased from Sigma-Aldrich. Rabbit polyclonal anti-TFEB (RRID:AB_11220225) was purhcased from Cell Signaling Technology (Leiden, The Netherlands). Phalloidin-iFluor 405 was obtained from Abcam (Cambridge, UK). Goat anti-rabbit IgG (H+L) AlexaFluor647 conjugate (RRID:AB_2536101) was purchased from Thermo Fisher Scientific (Breda, The Netherlands). All compounds, except gentamicin sulfate and hygromycin B, were dissolved in 100% dimethyl sulfoxide (DMSO; Sigma-Aldrich) in stock concentrations of 10 mM, aliquoted, and kept at −80°C.

### Primary macrophage culture.

Buffy coats were obtained from healthy donors after written informed consent (Sanquin Blood Bank, Amsterdam, The Netherlands). Peripheral blood mononuclear cells (PBMCs) were purified using density gradient centrifugation over Ficoll-Paque, and monocytes were isolated with subsequent CD14 magnetic activated cell sorting (MACS; Miltenyi Biotec, Bergisch Gladsbach, Germany) as described previously (35, 36). Monocytes were then differentiated into proinflammatory (Mφ1) or anti-inflammatory (Mφ2) macrophages with 5 ng/mL granulocyte-macrophage colony-stimulating factor (GM-CSF; Life Technologies-Invitrogen) or 50 ng/mL macrophage colony-stimulating factor (M-CSF; R&D Systems, Abingdon, UK), respectively, for 6 days with a cytokine boost at 3 days, as previously reported (45). Cells were cultured at a density of 1 × 10^6^ cells per mL in T75 flasks at 37°C and 5% CO_2_ in complete Roswell Park Memorial Institute (RPMI) composed of Gibco RPMI 1640 medium or RPMI 1640 (Dutch modified; Life Technologies-Invitrogen) supplemented with 10% fetal bovine serum (FBS) from Greiner Bio-One (Alphen aan den Rijn, The Netherlands), 2 mM l-alanyl-l-glutamine (GlutaMAX; PAA, Linz, Austria), and 100 U/mL penicillin and 100 μg/mL streptomycin (both Life Technologies-Invitrogen). Macrophages were harvested using 0.05% trypsin-EDTA (Thermo Fisher Scientific) and scraping. Macrophage differentiation was evaluated by quantification of interleukin-10 (IL-10) and IL-12p40 secretion using enzyme-linked immunosorbent assay (ELISA) in the presence or absence of 24-h stimulation with 100 ng/mL lipopolysaccharide (LPS; InvivoGen, San Diego, USA).

### Zebrafish culture.

Zebrafish were maintained and handled in compliance with the local animal welfare regulations as overseen by the Animal Welfare Body of Leiden University (license number 10612). All practices involving zebrafish were performed in accordance with European laws, guidelines, and policies for animal experimentation, housing, and care (European Directive 2010/63/EU on the protection of animals used for scientific purposes). The present study did not involve any procedures within the meaning of Article 3 of Directive 2010/63/EU and, as such, is not subject to authorization by an ethics committee. Zebrafish lines (Table S5 in the supplemental material) were maintained according to standard protocols (www.zfin.org). Zebrafish eggs were obtained by natural spawning of single crosses to achieve synchronized developmental timing. Eggs from at least five couples were combined to achieve heterogeneous groups. Eggs and embryos were kept in egg water (60 μg/mL sea salt; Sera Marin, Heinsberg, Germany) at ~28.5°C after harvesting and in embryo medium after infection and/or treatment (E2, buffered medium [15 mM NaCl, 0.5 mM KCl, 1 mM MgSO_4_, 150 μM KH_2_PO_4_, 1 mM CaCl_2_, and 0.7 mM NaHCO_3_]) at ~28.5°C for the duration of the experiments.

10.1128/mbio.03024-22.10 TABLE S5 Supplemental materials. Download Table S5, PDF file, 0.1 MB. Copyright © 2022 Boland et al. 2022 Boland et al. https://creativecommons.org/licenses/by/4.0/ This content is distributed under the terms of the Creative Commons Attribution 4.0 International license.

### Zebrafish genotyping.

Larvae obtained by incrossing heterozygous *esr2b*-mutant (*esr2_b_^+/−^*) zebrafish were genotyped at the endpoint of the infection experiments. Larvae were collected individually in 100 μL of 50 mM NaOH. Samples were heated to 95°C for 10 min until the larvae dissolved and cooled to 4°C. Then, 10 μL of 1 M Tris (pH 7.5) was added to neutralize the basic solution, and samples were centrifuged to pull down any tissue debris, essentially as described previously (85). Supernatant was directly used for PCR amplification of the genetic region of interest followed by Sanger sequencing to identify the genotype (BaseClear, The Netherlands). Sequences of the primers are provided in Table S5.

### Bacterial cultures.

*Mtb* (H37Rv), DsRed-expressing H37Rv, and mWasabi- or mCherry-expressing *Mm* M-strain (86, 87) were cultured in Difco Middlebrook 7H9 broth (Becton, Dickinson, Breda, The Netherlands) supplemented with 10% ADC (Becton, Dickinson), 0.05% Tween 80 (Sigma-Aldrich), and 50 μg/mL hygromycin B (Life Technologies-Invitrogen). *Stm* strain SL1344 was cultured in Difco lysogeny broth (LB; Becton, Dickinson). *Mtb* and *Stm* were cultured at 37°C, while *Mm* was grown at ~28.5°C.

### Bacterial infection of human cells.

*Mtb* and *Mm* suspensions were prepared from running cultures, which were diluted to a density corresponding with early log-phase growth (OD_550/600_ of 0.25) 1 day before infection. *Stm* was grown overnight, subsequently diluted 1:33 in fresh LB, and used after approximately 3 h of incubation when log-phase growth was achieved (OD_600_ of 0.5). Bacteria were diluted in complete RPMI or complete Iscove’s Modified Dulbecco’s Medium (IMDM) without antibiotics for infection of primary cells and MelJuSo cells, respectively, as described previously (35, 36). We consistently used a multiplicity of infection (MOI) of 10 for all *in vitro* infection experiments. Primary cells and MelJuSo cells were seeded at a density of 30,000 and 10,000 cells per well, respectively, in 96-well flat-bottom plates 1 day before infection and were inoculated with 100 μL of the bacterial suspension. Cells were subsequently centrifuged for 3 min at 800 rpm and incubated at 37°C and 5% CO_2_ for 20 min for *Stm* infections or 60 min for *Mtb* and *Mm* infections. Extracellular bacteria were then washed away with culture medium containing 30 μg/mL gentamicin sulfate and incubated for 10 min at 37°C and 5% CO_2_, followed by replacement with medium containing 5 μg/mL gentamicin sulfate and, if indicated, chemical compounds until readout. The MOI of the inoculum was verified by a standard CFU assay.

### Bacterial infection of zebrafish embryos.

Fresh *Mm* inoculum was prepared for every infection experiment as described previously (88). The final inoculum was resuspended in phosphate-buffered saline (PBS) containing 2% (wt/vol) polyvinylpyrrolidone (PVP40). The injection dose was determined by optical density measurement (an OD_600_ of 1 corresponds to ~100 CFU/nL). Infection experiments were performed according to previously described procedures (88). In brief, microinjections were performed using borosilicate glass microcapillary injection needles (Harvard Apparatus, 300038, 1-mm outside diameter [o.d.] × 0.78-mm inside diameter [i.d.]) prepared using a micropipette puller device (Sutter Instruments Flaming/Brown P-97). Needles were mounted on a micromanipulator (Sutter Instruments, MM-33R) positioned under a stereomicroscope. Before injection at 30 h postfertilization (hpf), embryos were anesthetized using 200 μg/mL buffered 3-aminobenzoid acid (tricaine; Sigma-Aldrich) in egg water. Embryos were then positioned on a 1% agarose plate (in egg water) and injected into the blood island with 1 nL of inoculum containing ~200 CFU *Mm*. For assessment of bacterial burden, larvae were anesthetized using tricaine at 4 days postinfection (dpi), positioned on a 1% agarose (in egg water) plate, and imaged using a Leica M205 FA fluorescence stereomicroscope equipped with a DFC345 FX monochrome camera. Bacterial burden was determined based on fluorescent pixel quantification (89).

### Chemical compound treatments.

Cells were treated with chemical compound or 100% DMSO at equal volumes in medium containing 5 μg/mL gentamicin sulfate as described previously (35, 36). Treatment of zebrafish embryos was performed by immersion. Stock concentrations were diluted to treatment doses in complete IMDM or embryo medium without antibiotics for human cells and zebrafish embryos, respectively. As a solvent control treatment, 100% DMSO was diluted to the same concentration as the compound treatment. If different tamoxifen treatment doses were used in the same zebrafish embryo experiment, the solvent control concentration corresponding to the highest tamoxifen treatment dose was used. Precise doses of compound treatments and solvent control concentrations as well as the durations of treatment are described in the figure legends for each individual experiment.

### CFU assay.

Cells were lysed in water containing 0.05% SDS (Thermo Fisher Scientific). Lysates of *Mtb-*infected cells were serially diluted 5-fold in 7H9 broth, and 10-μL droplets were spotted onto square Middlebrook 7H10 agar plates. Plates were incubated at 37°C for 12 to 14 days, and bacterial colonies were quantified using a microscope at a ×2.5 magnification to enhance early detection of bacterial growth. Lysates of *Stm*-infected cells were serially diluted in 10-fold steps in LB broth, and 10-μL droplets were spotted onto square LB agar plates and incubated overnight at 37°C.

### Liquid bacterial growth assay.

*Stm* or *Mtb* and *Mm* cultures in logarithmic growth phase were diluted to an OD_550_ or OD_600_ of 0.05 in LB broth or 0.1 in complete 7H9 broth, respectively, of which, 200 μL per well in a flat-bottom 96-well plate was incubated with chemical compound, antibiotic, or DMSO at equal volumes at the indicated concentrations. *Stm* growth (OD_550_) was measured after overnight incubation at 37°C, while *Mm* growth was evaluated during 2 days of incubation at ~28.5°C and *Mtb* growth for 10 days of incubation at 37°C.

### Lactate dehydrogenase (LDH) release assay.

At the experimental endpoint, primary cells, infected and treated as described above, were centrifuged for 3 min at 800 rpm. Eighty microliters of supernatant was transferred to a flat-bottom 96-well plate, and 100 μL of freshly prepared reaction mixture (250 μL of reconstituted catalyst plus 11.25 mL of dye solution; cytotoxicity detection kit LDH, Roche) was added. After a 30-min incubation at room temperature (RT) protected from light, absorbance at OD_485_ and OD_690_ was measured using an Envision plate reader (PerkinElmer, Rotterdam, the Netherlands). Toxicity was calculated using the formula ([sample value − spontaneous release]/[maximum release value − spontaneous release], where spontaneous release indicates untreated cells, and maximum release indicates cells lysed using 2% Triton X-100).

### Flow cytometry.

At the experimental endpoint, infected cells were washed with 100 μL of PBS and detached by incubation in 50 μL of 0.05% trypsin-EDTA for several minutes. Single-cell suspensions were fixed by adding 100 μL of 1.5% paraformaldehyde with subsequent incubation for 60 min at 4°C. Acquisition was performed using a BD FACSCalibur combined with a high-throughput sampler (HTS; BD BioSciences). Data were analyzed using FlowJo software v9.

### Immunostaining.

Cells were seeded on poly-d-lysine-coated glass-bottom (number 1.5; prewashed with PBS) 96-well plates (MatTek, Ashland, MA, USA) at a density of 30,000 cells per well. After overnight incubation, cells were infected with DsRed-expressing *Mtb* at an MOI of 10 as described above. At the indicated experimental endpoint, cells were washed three times with PBS and fixed for 60 min at RT using 1% methanol-free electron microscopy (EM)-grade formaldehyde (Thermo Fisher Scientific) diluted in PBS. Cells were washed with PBS, and remaining reactive formaldehyde was quenched using 100 μL of glycine solution (1.5 mg/mL in PBS) for 10 min at RT. Fluorescent dyes LysoTracker Deep Red (75 nM; Thermo Fisher Scientific) and Cyto-ID 2.0 (1:500; Enzo LifeSciences) were added to the cells 30 min before the treatment endpoint, and, after the washing and fixation procedure described above, cells were counterstained with 50 μL of 2 μg/mL Hoechst 33342 (Sigma-Aldrich) at RT in the dark. For staining using antibodies, cells were permeabilized in 0.1% Triton-X (Sigma-Aldrich) diluted in PBS for 10 min at RT, and Fc receptors were subsequently blocked using 5% human serum (HS; Sanquin Blood Bank, Amsterdam, The Netherlands) for 45 min at RT. After removal of the 5% HS, cells were stained with 50 μL of primary antibody diluted in 5% HS for 30 min at RT, washed three times with 5% HS, and incubated with 50 μL of secondary antibody in 5% HS for 30 min at RT in the dark. After washing three times with 5% HS, cells were counterstained with Hoechst 33342 and phalloidin as described above. Images (at least three per well) were acquired using a Leica TCS SP8 X WLL confocal system and a ×63 oil immersion objective. Hybrid detectors were used with a time gate to switch off data collection during the pulse.

Colocalization analysis of *Mtb*-infected cells was performed using the following procedure. Image background was subtracted using the rolling ball (20-pixel radius) algorithm with Fiji software version 1.53c (90). CellProfiler 3.0.0 (91) was used to first correct for nonhomogenous illumination if necessary and for the segmentation of both the fluorescent bacteria and marker of interest using global thresholding with intensity-based declumping (91). For every experiment, segmentation was performed with both a range of thresholds and adaptive three-class Otsu thresholding independently to confirm segmentation results. Then, per image, the overlap of *Mtb* with the marker of interest was calculated as percentage of object overlap. To avoid potentially confounding results, two donors were excluded from colocalization results (Fig. 4A and 5B) due to extensive intensity background levels in treated samples.

### GFP-Lc3 and LysoTracker imaging in zebrafish larvae.

For visualization of Lc3 dynamics, Tg(CMV:EGFP-map1lc3b) larvae were embedded in 1.5% low-melting-point agarose (weight per volume, in egg water) and imaged using a Leica TCS SPE confocal microscope. Imaging was performed using a ×63 oil immersion objective (HC PL APO CS2, numerical aperture [NA] of 1.42) in a region of the tail fin to detect enhanced GFP (EGFP)-map1lc3b (GFP-Lc3)-positive vesicles. For quantification of acidic vesicles in the presence and absence of infection, larvae were immersed in embryo medium containing 5 μM LysoTracker Red DND-99 solution (Thermo Fisher Scientific) for 1 h. Before mounting and imaging, larvae were washed three times with embryo medium. To determine colocalization between *Mm* and GFP-Lc3 or LysoTracker, fixed (GFP-Lc3) or live anesthetized (LysoTracker) larvae were embedded in 1.5% low-melting-point agarose (in egg water) and imaged in the caudal hematopoietic tissue using a Leica TCS SP8 confocal microscope with a ×40 water immersion objective (HCX APO L U-V-I, NA of 0.8). Images were obtained using Leica Las X software. For the quantification of GFP-Lc3 levels, the find maxima algorithm with a noise tolerance of 50 was used in Fiji software version 1.53c. To determine association of GFP-Lc3 or LysoTracker with bacteria, manual counting was performed on the obtained confocal images using Leica Las X software.

### Tail amputation of zebrafish larvae.

Embryos of an Tg(mpeg1:mcherryF)/Tg(mpx;gfp) double transgenic line were anesthetized using tricaine at 3 days postfertilization (dpf) and positioned on a 1% agarose (in egg water) plate, and the tails were partially amputated with a 1-mm sapphire blade (World Precision Instruments) under a Leica M165C stereomicroscope (92). After amputation, larvae were incubated in embryo medium for 4 h and fixed using 4% paraformaldehyde. After fixation, larvae were positioned on a 1% agarose (in egg water) plate and imaged using a Leica M205 FA fluorescence stereomicroscope equipped with a DFC345 FX monochrome camera. Macrophages were detected based on the fluorescence of their mCherry label, and neutrophils were detected based on their GFP label. The number of leukocytes recruited to the wounded area were counted as described previously (92).

### RNA isolation, cDNA synthesis, and qPCR.

Zebrafish larvae (10 per sample) were collected at the experimental endpoint in QIAzol lysis reagent (Qiagen, Hilden, Germany). RNA was isolated using an miRNeasy minikit (Qiagen, Hilden, Germany) according to the manufacturer’s instructions, and the iScript cDNA synthesis kit (Bio-Rad, Hercules, USA) was used for reverse transcription of the extracted total RNA. qPCR was performed on a Bio-Rad CFX96 machine following a two-step protocol with 40 cycles, with a 95°C melting temperature for 15 s and a 60°C annealing temperature and amplification for 30 s. All reactions on the 3 biological replicates (3 samples/treatment group) were performed with 3 technical replicates (3 wells/sample). Analysis of qPCR results was performed using the cycling threshold (ΔΔ*C_T_*) method, and data were normalized to the expression of the housekeeping gene *tbp* (TATA box binding protein). Two ER target genes were analyzed: *cyp19a1b* (cytochrome P450, family 19, subfamily A, polypeptide 1a) and *vtg1* (vitellogenin 1). Sequences of the primers are provided in Table S5.

### RNA sequencing and data analysis.

Tamoxifen treatment of zebrafish larvae was performed from 1 h postinfection (hpi) until 2 dpi (3 dpf). Next, larvae were collected (10 per sample) for RNA isolation as described above. RNA integrity was assessed by Bioanalyzer (Agilent, Santa Clara, USA), and all samples were found to have an RNA integrity number (RIN) of ≥9.5. Of the total RNA, 3 μg was used to create RNA-sequencing libraries using the Illumina TruSeq strand-specific mRNA poly(A) preparation kit (Illumina, San Diego, USA). The resulting RNA-sequencing library was sequenced for at least 10 million reads per sample using an Illumina HiSeq2500 with a read length of 1 × 50 nucleotides (Baseclear, Leiden, The Netherlands). Four biological replicates for each treatment and infection regimen were sequenced and mapped and quantified against the Danio rerio GRCzv11 using Salmon v0.14.1 (93). Downstream analysis of the quantified libraries was performed in RStudio 1.2.5001 (94) running R 3.6.1 (95). Libraries were imported using tximport v.1.12.3 (96). Differential gene expression was assessed via pairwise comparisons using DESeq2 v1.24.0 (97) following a linear model, taking into account possible gene expression differences from the embryo parents, drug treatments, infections, and their interaction (design: ~genotype + treatment + infection + treatment:infection). Statistical significance was defined by an *S* value of ≤0.005 using apeglm (98). *S* values are aggregate statistics that have been recently proposed as an alternative to adjusted *P* values and false-discovery rates (FDRs), calculating the probability of getting the sign of an effect wrong in biological contexts (99).

Venn diagram generation and enrichment analyses, including pathway and GO analyses as well as gene set enrichment analysis with the C2 “Curated Gene Sets” and C5 “GO Gene Sets” collections from the Molecular Signatures Database (MSigDB), were performed as previously described (44).

### Data analysis and statistics.

An unpaired *t* test or one-way analysis of variance (ANOVA) with Dunnett’s or Hold-Sidak’s multiple-comparison test was applied when assessing differences between two or more groups, respectively, of unpaired data representing technical replicates. A Mann-Whitney *U* test was applied for testing differences between unpaired data representing biological replicates. When assessing differences between two or more groups with paired observations of biological replicates, the Wilcoxon matched-pairs signed-rank test was used, except for data that was normally distributed according to the Shapiro-Wilk test, which was assessed using a (ratio) paired *t* test or repeated measures (RM) one-way ANOVA with a Holm-Sidak multiple-testing correction. A two-way ANOVA with Dunnett’s or Tukey’s multiple-comparison test was used when the effect of two independent variables was tested simultaneously to either a control mean or every other mean of data representing technical replicates, respectively. Data were normalized to the mean of the control group, and independent repeats were combined, unless otherwise indicated. The number of experiments combined is indicated in the figure legend for each experiment. With the exception of the transcriptome profiling analysis, all analyses were performed using GraphPad Prism 8, and the statistical tests performed for each experiment are described in the figure legends. Dot plot graphs of zebrafish experiments were made using the raincloud plots application at https://gabrifc.shinyapps.io/raincloudplots/ (100). All data shown have been subjected to statistical testing; comparisons that are not significantly different lack any indication.

### Data availability.

Raw data are deposited at the Gene Expression Omnibus under accession number GSE178919. The data and code to recapitulate all figures and findings in the manuscript are available at https://github.com/gabrifc/analysis-tamox-transcriptome.

## References

[1] Houben RMGJ, Dodd PJ. 2016. The global burden of latent tuberculosis infection: a re-estimation using mathematical modelling. PLoS Med 13:e1002152. doi:10.1371/journal.pmed.1002152. 27780211 PMC5079585

[2] WHO. 2020. Global tuberculosis report 2020. World Health Organization, Geneva, Switzerland.

[3] Davenne T, McShane H. 2016. Why don’t we have an effective tuberculosis vaccine yet? Expert Rev Vaccines 15:1009–1013. doi:10.1586/14760584.2016.1170599. 27010255 PMC4950406

[4] Diacon AH, Donald PR, Pym A, Grobusch M, Patientia RF, Mahanyele R, Bantubani N, Narasimooloo R, De Marez T, van Heeswijk R, Lounis N, Meyvisch P, Andries K, McNeeley DF. 2012. Randomized pilot trial of eight weeks of bedaquiline (TMC207) treatment for multidrug-resistant tuberculosis: long-term outcome, tolerability, and effect on emergence of drug resistance. Antimicrob Agents Chemother 56:3271–3276. doi:10.1128/AAC.06126-11. 22391540 PMC3370813

[5] Gler MT, Skripconoka V, Sanchez-Garavito E, Xiao H, Cabrera-Rivero JL, Vargas-Vasquez DE, Gao M, Awad M, Park SK, Shim TS, Suh GY, Danilovits M, Ogata H, Kurve A, Chang J, Suzuki K, Tupasi T, Koh WJ, Seaworth B, Geiter LJ, Wells CD. 2012. Delamanid for multidrug-resistant pulmonary tuberculosis. N Engl J Med 366:2151–2160. doi:10.1056/NEJMoa1112433. 22670901

[6] Lee M, Lee J, Carroll MW, Choi H, Min S, Song T, Via LE, Goldfeder LC, Kang E, Jin B, Park H, Kwak H, Kim H, Jeon HS, Jeong I, Joh JS, Chen RY, Olivier KN, Shaw PA, Follmann D, Song SD, Lee JK, Lee D, Kim CT, Dartois V, Park SK, Cho SN, Barry CE, III. 2012. Linezolid for treatment of chronic extensively drug-resistant tuberculosis. N Engl J Med 367:1508–1518. doi:10.1056/NEJMoa1201964. 23075177 PMC3814175

[7] Keam SJ. 2019. Pretomanid: first approval. Drugs 79:1797–1803. doi:10.1007/s40265-019-01207-9. 31583606

[8] Kadura S, King N, Nakhoul M, Zhu H, Theron G, Koser CU, Farhat M. 2020. Systematic review of mutations associated with resistance to the new and repurposed *Mycobacterium tuberculosis* drugs bedaquiline, clofazimine, linezolid, delamanid and pretomanid. J Antimicrob Chemother 75:2031–2043. doi:10.1093/jac/dkaa136. 32361756 PMC7825472

[9] Vergne I, Gilleron M, Nigou J. 2014. Manipulation of the endocytic pathway and phagocyte functions by *Mycobacterium tuberculosis* lipoarabinomannan. Front Cell Infect Microbiol 4:187. doi:10.3389/fcimb.2014.00187. 25629008 PMC4290680

[10] Bussi C, Gutierrez MG. 2019. *Mycobacterium tuberculosis* infection of host cells in space and time. FEMS Microbiol Rev 43:341–361. doi:10.1093/femsre/fuz006. 30916769 PMC6606852

[11] Machelart A, Song OR, Hoffmann E, Brodin P. 2017. Host-directed therapies offer novel opportunities for the fight against tuberculosis. Drug Discov Today 22:1250–1257. doi:10.1016/j.drudis.2017.05.005. 28533187

[12] Kilinc G, Saris A, Ottenhoff THM, Haks MC. 2021. Host-directed therapy to combat mycobacterial infections*. Immunol Rev 301:62–83. doi:10.1111/imr.12951. 33565103 PMC8248113

[13] Wallis RS, Hafner R. 2015. Advancing host-directed therapy for tuberculosis. Nat Rev Immunol 15:255–263. doi:10.1038/nri3813. 25765201

[14] Zumla A, Maeurer M, Host-Directed Therapies Network, Chakaya J, Hoelscher M, Ntoumi F, Rustomjee R, Vilaplana C, Yeboah-Manu D, Rasolof V, Munderi P, Singh N, Aklillu E, Padayatchi N, Macete E, Kapata N, Mulenga M, Kibiki G, Mfinanga S, Nyirenda T, Maboko L, Garcia-Basteiro A, Rakotosamimanana N, Bates M, Mwaba P, Reither K, Gagneux S, Edwards S, Mfinanga E, Abdulla S, Cardona P-J, Russell JBW, Gant V, Noursadeghi M, Elkington P, Bonnet M, Menendez C, Dieye TN, Diarra B, Maiga A, Aseffa A, Parida S, Wejse C, Petersen E, Kaleebu P, Oliver M, Craig G, Corrah T, Tientcheu L, Antonio M, Rao M, et al. 2015. Towards host-directed therapies for tuberculosis. Nat Rev Drug Discov 14:511–512. doi:10.1038/nrd4696. 26184493

[15] Hawn TR, Matheson AI, Maley SN, Vandal O. 2013. Host-directed therapeutics for tuberculosis: can we harness the host? Microbiol Mol Biol Rev 77:608–627. doi:10.1128/MMBR.00032-13. 24296574 PMC3973381

[16] Deretic V, Saitoh T, Akira S. 2013. Autophagy in infection, inflammation and immunity. Nat Rev Immunol 13:722–737. doi:10.1038/nri3532. 24064518 PMC5340150

[17] Maiuri MC, Kroemer G. 2019. Therapeutic modulation of autophagy: which disease comes first? Cell Death Differ 26:680–689. doi:10.1038/s41418-019-0290-0. 30728461 PMC6460393

[18] Keller MD, Torres VJ, Cadwell K. 2020. Autophagy and microbial pathogenesis. Cell Death Differ 27:872–886. doi:10.1038/s41418-019-0481-8. 31896796 PMC7205878

[19] Kimmey JM, Stallings CL. 2016. Bacterial pathogens versus autophagy: implications for therapeutic interventions. Trends Mol Med 22:1060–1076. doi:10.1016/j.molmed.2016.10.008. 27866924 PMC5215815

[20] Gutierrez MG, Master SS, Singh SB, Taylor GA, Colombo MI, Deretic V. 2004. Autophagy is a defense mechanism inhibiting BCG and *Mycobacterium tuberculosis* survival in infected macrophages. Cell 119:753–766. doi:10.1016/j.cell.2004.11.038. 15607973

[21] Bradfute SB, Castillo EF, Arko-Mensah J, Chauhan S, Jiang S, Mandell M, Deretic V. 2013. Autophagy as an immune effector against tuberculosis. Curr Opin Microbiol 16:355–365. doi:10.1016/j.mib.2013.05.003. 23790398 PMC3742717

[22] Tobin DM. 2015. Host-directed therapies for tuberculosis. Cold Spring Harb Perspect Med 5:a021196. doi:10.1101/cshperspect.a021196. 25986592 PMC4588138

[23] Zembutsu H. 2015. Pharmacogenomics toward personalized tamoxifen therapy for breast cancer. Pharmacogenomics 16:287–296. doi:10.2217/pgs.14.171. 25712191

[24] Gallo MA, Kaufman D. 1997. Antagonistic and agonistic effects of tamoxifen: significance in human cancer. Semin Oncol 24:S1-71–S1-80. 9045319

[25] Ahmad I. 2018. Tamoxifen a pioneering drug: an update on the therapeutic potential of tamoxifen derivatives. Eur J Med Chem 143:515–531. doi:10.1016/j.ejmech.2017.11.056. 29207335

[26] Heemskerk MK, Korbee CJ, Esselink J, Carvalho dos Santos C, van Veen S, Gordijn IF, Vrieling F, Walburg KV, Engele CG, Dijkman D, Wilson L, Verreck FAW, Ottenhoff THM, Haks MC. 2021. Repurposing diphenylbutylpiperidine-class antipsychotic drugs for host-directed therapy of *Mycobacterium tuberculosis* and *Salmonella enterica* infections. Sci Rep 11:19634. doi:10.1101/2021.06.05.447191. 34608194 PMC8490354

[27] Miguel DC, Zauli-Nascimento RC, Yokoyama-Yasunaka JKU, Katz S, Barbieri CL, Uliana SRB. 2009. Tamoxifen as a potential antileishmanial agent: efficacy in the treatment of *Leishmania braziliensis* and *Leishmania chagasi* infections. J Antimicrob Chemother 63:365–368. doi:10.1093/jac/dkn509. 19095684

[28] Butts A, Koselny K, Chabrier-Rosello Y, Semighini CP, Brown JC, Wang X, Annadurai S, DiDone L, Tabroff J, Childers WE, Jr, Abou-Gharbia M, Wellington M, Cardenas ME, Madhani HD, Heitman J, Krysan DJ. 2014. Estrogen receptor antagonists are anti-cryptococcal agents that directly bind EF hand proteins and synergize with fluconazole *in vivo*. mBio 5:e00765-13. doi:10.1128/mBio.00765-13. 24520056 PMC3950514

[29] Chen FC, Liao YC, Huang JM, Lin CH, Chen YY, Dou HY, Hsiung CA. 2014. Pros and cons of the tuberculosis drugome approach—an empirical analysis. PLoS One 9:e100829. doi:10.1371/journal.pone.0100829. 24971632 PMC4074101

[30] Jang WS, Kim S, Podder B, Jyoti MA, Nam KW, Lee BE, Song HY. 2015. Anti-mycobacterial activity of tamoxifen against drug-resistant and intra-macrophage Mycobacterium tuberculosis. J Microbiol Biotechnol 25:946–950. doi:10.4014/jmb.1412.12023. 25639719

[31] Dittmar AJ, Drozda AA, Blader IJ. 2016. Drug repurposing screening identifies novel compounds that effectively inhibit *Toxoplasma gondii* growth. mSphere 1:e00042-15 doi:10.1128/mSphere.00042-15. PMC4894684 27303726

[32] Corleis B, Dorhoi A. 2020. Early dynamics of innate immunity during pulmonary tuberculosis. Immunol Lett 221:56–60. doi:10.1016/j.imlet.2020.02.010. 32092359

[33] Rothchild AC, Olson GS, Nemeth J, Amon LM, Mai D, Gold ES, Diercks AH, Aderem A. 2019. Alveolar macrophages generate a noncanonical NRF2-driven transcriptional response to *Mycobacterium tuberculosis in vivo*. Sci Immunol 4:eaaw6693. doi:10.1126/sciimmunol.aaw6693. 31350281 PMC6910245

[34] Thiriot JD, Martinez-Martinez YB, Endsley JJ, Torres AG. 2020. Hacking the host: exploitation of macrophage polarization by intracellular bacterial pathogens. Pathog Dis 78:ftaa009. doi:10.1093/femspd/ftaa009. 32068828 PMC7069348

[35] Korbee CJ, Heemskerk MT, Kocev D, van Strijen E, Rabiee O, Franken K, Wilson L, Savage NDL, Dzeroski S, Haks MC, Ottenhoff THM. 2018. Combined chemical genetics and data-driven bioinformatics approach identifies receptor tyrosine kinase inhibitors as host-directed antimicrobials. Nat Commun 9:358. doi:10.1038/s41467-017-02777-6. 29367740 PMC5783939

[36] Moreira JD, Koch BEV, van Veen S, Walburg KV, Vrieling F, Guimaraes TMPD, Meijer AH, Spaink HP, Ottenhoff THM, Haks MC, Heemskerk MT. 2020. Functional inhibition of host histone deacetylases (HDACs) enhances *in vitro* and *in vivo* anti-mycobacterial activity in human macrophages and in zebrafish. Front Immunol 11:36. doi:10.3389/fimmu.2020.00036. 32117228 PMC7008710

[37] Keiser TL, Purdy GE. 2017. Killing *Mycobacterium tuberculosis* in vitro: what model systems can teach us. Microbiol Spectr 5. doi:10.1128/microbiolspec.TBTB2-0028-2016. PMC6714986 28597814

[38] Davis JM, Clay H, Lewis JL, Ghori N, Herbomel P, Ramakrishnan L. 2002. Real-time visualization of mycobacterium-macrophage interactions leading to initiation of granuloma formation in zebrafish embryos. Immunity 17:693–702. doi:10.1016/s1074-7613(02)00475-2. 12479816

[39] Meijer AH. 2016. Protection and pathology in TB: learning from the zebrafish model. Semin Immunopathol 38:261–273. doi:10.1007/s00281-015-0522-4. 26324465 PMC4779130

[40] Berg RD, Ramakrishnan L. 2012. Insights into tuberculosis from the zebrafish model. Trends Mol Med 18:689–690. doi:10.1016/j.molmed.2012.10.002. 23084762

[41] van der Vaart M, Korbee CJ, Lamers GE, Tengeler AC, Hosseini R, Haks MC, Ottenhoff TH, Spaink HP, Meijer AH. 2014. The DNA damage-regulated autophagy modulator DRAM1 links mycobacterial recognition via TLR-MYD88 to autophagic defense [corrected]. Cell Host Microbe 15:753–767. doi:10.1016/j.chom.2014.05.005. 24922577

[42] Hosseini R, Lamers GE, Hodzic Z, Meijer AH, Schaaf MJ, Spaink HP. 2014. Correlative light and electron microscopy imaging of autophagy in a zebrafish infection model. Autophagy 10:1844–1857. doi:10.4161/auto.29992. 25126731 PMC4198367

[43] Zhang R, Varela M, Vallentgoed W, Forn-Cuni G, van der Vaart M, Meijer AH. 2019. The selective autophagy receptors optineurin and p62 are both required for zebrafish host resistance to mycobacterial infection. PLoS Pathog 15:e1007329. doi:10.1371/journal.ppat.1007329. 30818338 PMC6413957

[44] Zhang R, Varela M, Forn-Cuni G, Torraca V, van der Vaart M, Meijer AH. 2020. Deficiency in the autophagy modulator Dram1 exacerbates pyroptotic cell death of *Mycobacteria*-infected macrophages. Cell Death Dis 11:277. doi:10.1038/s41419-020-2477-1. 32332700 PMC7181687

[45] Verreck FAW, de Boer T, Langenberg DML, van der Zanden L, Ottenhoff THM. 2006. Phenotypic and functional profiling of human proinflammatory type-1 and anti-inflammatory type-2 macrophages in response to microbial antigens and IFN-gamma- and CD40L-mediated costimulation. J Leukoc Biol 79:285–293. doi:10.1189/jlb.0105015. 16330536

[46] Verreck FA, de Boer T, Langenberg DM, Hoeve MA, Kramer M, Vaisberg E, Kastelein R, Kolk A, de Waal-Malefyt R, Ottenhoff TH. 2004. Human IL-23-producing type 1 macrophages promote but IL-10-producing type 2 macrophages subvert immunity to (myco)bacteria. Proc Natl Acad Sci USA 101:4560–4565. doi:10.1073/pnas.0400983101. 15070757 PMC384786

[47] Benard EL, Rougeot J, Racz PI, Spaink HP, Meijer AH. 2016. Transcriptomic approaches in the zebrafish model for tuberculosis—insights into host- and pathogen-specific determinants of the innate immune response. Adv Genet 95:217–251. doi:10.1016/bs.adgen.2016.04.004. 27503359

[48] Cardoso CM, Custodio JB, Almeida LM, Moreno AJ. 2001. Mechanisms of the deleterious effects of tamoxifen on mitochondrial respiration rate and phosphorylation efficiency. Toxicol Appl Pharmacol 176:145–152. doi:10.1006/taap.2001.9265. 11714246

[49] Nazarewicz RR, Zenebe WJ, Parihar A, Larson SK, Alidema E, Choi J, Ghafourifar P. 2007. Tamoxifen induces oxidative stress and mitochondrial apoptosis via stimulating mitochondrial nitric oxide synthase. Cancer Res 67:1282–1290. doi:10.1158/0008-5472.CAN-06-3099. 17283165

[50] Pattingre S, Bauvy C, Levade T, Levine B, Codogno P. 2009. Ceramide-induced autophagy: to junk or to protect cells? Autophagy 5:558–560. doi:10.4161/auto.5.4.8390. 19337026 PMC3501009

[51] Davis JM, Ramakrishnan L. 2009. The role of the granuloma in expansion and dissemination of early tuberculous infection. Cell 136:37–49. doi:10.1016/j.cell.2008.11.014. 19135887 PMC3134310

[52] Yang CT, Cambier CJ, Davis JM, Hall CJ, Crosier PS, Ramakrishnan L. 2012. Neutrophils exert protection in the early tuberculous granuloma by oxidative killing of mycobacteria phagocytosed from infected macrophages. Cell Host Microbe 12:301–312. doi:10.1016/j.chom.2012.07.009. 22980327 PMC3638950

[53] Torraca V, Cui C, Boland R, Bebelman JP, van der Sar AM, Smit MJ, Siderius M, Spaink HP, Meijer AH. 2015. The CXCR3-CXCL11 signaling axis mediates macrophage recruitment and dissemination of mycobacterial infection. Dis Model Mech 8:253–269. doi:10.1242/dmm.017756. 25573892 PMC4348563

[54] Corriden R, Hollands A, Olson J, Derieux J, Lopez J, Chang JT, Gonzalez DJ, Nizet V. 2015. Tamoxifen augments the innate immune function of neutrophils through modulation of intracellular ceramide. Nat Commun 6:8369. doi:10.1038/ncomms9369. 26458291 PMC4610010

[55] Ligeiro de Oliveira AP, Oliveira-Filho RM, da Silva ZL, Borelli P, Tavares de Lima W. 2004. Regulation of allergic lung inflammation in rats: interaction between estradiol and corticosterone. Neuroimmunomodulation 11:20–27. doi:10.1159/000072965. 14557675

[56] Moreland JG, Davis AP, Bailey G, Nauseef WM, Lamb FS. 2006. Anion channels, including ClC-3, are required for normal neutrophil oxidative function, phagocytosis, and transendothelial migration. J Biol Chem 281:12277–12288. doi:10.1074/jbc.M511030200. 16522634

[57] Xie Y, Meijer AH, Schaaf MJM. 2020. Modeling inflammation in zebrafish for the development of anti-inflammatory drugs. Front Cell Dev Biol 8:620984. doi:10.3389/fcell.2020.620984. 33520995 PMC7843790

[58] Dluzen DE, Mickley KR. 2005. Gender differences in modulatory effects of tamoxifen upon the nigrostriatal dopaminergic system. Pharmacol Biochem Behav 80:27–33. doi:10.1016/j.pbb.2004.10.007. 15652377

[59] Campesi I, Marino M, Montella A, Pais S, Franconi F. 2017. Sex differences in estrogen receptor alpha and beta levels and activation status in LPS-stimulated human macrophages. J Cell Physiol 232:340–345. doi:10.1002/jcp.25425. 27171902

[60] Subramanian A, Tamayo P, Mootha VK, Mukherjee S, Ebert BL, Gillette MA, Paulovich A, Pomeroy SL, Golub TR, Lander ES, Mesirov JP. 2005. Gene set enrichment analysis: a knowledge-based approach for interpreting genome-wide expression profiles. Proc Natl Acad Sci USA 102:15545–15550. doi:10.1073/pnas.0506580102. 16199517 PMC1239896

[61] Menuet A, Pellegrini E, Anglade I, Blaise O, Laudet V, Kah O, Pakdel F. 2002. Molecular characterization of three estrogen receptor forms in zebrafish: binding characteristics, transactivation properties, and tissue distributions. Biol Reprod 66:1881–1892. doi:10.1095/biolreprod66.6.1881. 12021076

[62] Griffin LB, January KE, Ho KW, Cotter KA, Callard GV. 2013. Morpholino-mediated knockdown of ERα, ERβa, and ERβb mRNAs in zebrafish (*Danio rerio*) embryos reveals differential regulation of estrogen-inducible genes. Endocrinology 154:4158–4169. doi:10.1210/en.2013-1446. 23928376 PMC3800766

[63] Lopez-Munoz A, Liarte S, Gomez-Gonzalez NE, Cabas I, Meseguer J, Garcia-Ayala A, Mulero V. 2015. Estrogen receptor 2b deficiency impairs the antiviral response of zebrafish. Dev Comp Immunol 53:55–62. doi:10.1016/j.dci.2015.06.008. 26133072

[64] Nagelkerke A, Bussink J, Sweep FCGJ, Span PN. 2014. The unfolded protein response as a target for cancer therapy. Biochim Biophys Acta 1846:277–284. doi:10.1016/j.bbcan.2014.07.006. 25069067

[65] He C, Bartholomew CR, Zhou W, Klionsky DJ. 2009. Assaying autophagic activity in transgenic GFP-Lc3 and GFP-Gabarap zebrafish embryos. Autophagy 5:520–526. doi:10.4161/auto.5.4.7768. 19221467 PMC2754832

[66] Settembre C, Di Malta C, Polito VA, Garcia Arencibia M, Vetrini F, Erdin S, Erdin SU, Huynh T, Medina D, Colella P, Sardiello M, Rubinsztein DC, Ballabio A. 2011. TFEB links autophagy to lysosomal biogenesis. Science 332:1429–1433. doi:10.1126/science.1204592. 21617040 PMC3638014

[67] Di Malta C, Cinque L, Settembre C. 2019. Transcriptional regulation of autophagy: mechanisms and diseases. Front Cell Dev Biol 7:114. doi:10.3389/fcell.2019.00114. 31312633 PMC6614182

[68] Ouyang Q, Zhang K, Lin D, Feng CG, Cai Y, Chen X. 2020. Bazedoxifene suppresses intracellular *Mycobacterium tuberculosis* growth by enhancing autophagy. mSphere 5:e00124-20. doi:10.1128/mSphere.00124-20. 32269154 PMC7142296

[69] Miro-Canturri A, Ayerbe-Algaba R, Del Toro R, Mejias ME, Pachon J, Smani Y. 2021. Potential tamoxifen repurposing to combat infections by multidrug-resistant Gram-negative bacilli. Pharmaceuticals (Basel) 14:507. doi:10.3390/ph14060507. 34073235 PMC8230278

[70] Hao R, Bondesson M, Singh AV, Riu A, McCollum CW, Knudsen TB, Gorelick DA, Gustafsson JA. 2013. Identification of estrogen target genes during zebrafish embryonic development through transcriptomic analysis. PLoS One 8:e79020. doi:10.1371/journal.pone.0079020. 24223173 PMC3819264

[71] Vosges M, Kah O, Hinfray N, Chadili E, Le Page Y, Combarnous Y, Porcher JM, Brion F. 2012. 17α-Ethinylestradiol and nonylphenol affect the development of forebrain GnRH neurons through an estrogen receptors-dependent pathway. Reprod Toxicol 33:198–204. doi:10.1016/j.reprotox.2011.04.005. 21549831

[72] Bao Y, Wang L, Sun J. 2021. A small protein but with diverse roles: a review of EsxA in *Mycobacterium*-host interaction. Cells 10:1645. doi:10.3390/cells10071645. 34209120 PMC8305481

[73] Wang J, Wang R, Wang H, Yang X, Yang J, Xiong W, Wen Q, Ma L. 2017. Glucocorticoids suppress antimicrobial autophagy and nitric oxide production and facilitate mycobacterial survival in macrophages. Sci Rep 7:982. doi:10.1038/s41598-017-01174-9. 28428627 PMC5430514

[74] Yang CS, Kim JJ, Lee HM, Jin HS, Lee SH, Park JH, Kim SJ, Kim JM, Han YM, Lee MS, Kweon GR, Shong M, Jo EK. 2014. The AMPK-PPARGC1A pathway is required for antimicrobial host defense through activation of autophagy. Autophagy 10:785–802. doi:10.4161/auto.28072. 24598403 PMC5119058

[75] Kimmey JM, Huynh JP, Weiss LA, Park S, Kambal A, Debnath J, Virgin HW, Stallings CL. 2015. Unique role for ATG5 in neutrophil-mediated immunopathology during *M. tuberculosis* infection. Nature 528:565–569. doi:10.1038/nature16451. 26649827 PMC4842313

[76] Oeste CL, Seco E, Patton WF, Boya P, Perez-Sala D. 2013. Interactions between autophagic and endo-lysosomal markers in endothelial cells. Histochem Cell Biol 139:659–670. doi:10.1007/s00418-012-1057-6. 23203316

[77] Ponpuak M, Davis AS, Roberts EA, Delgado MA, Dinkins C, Zhao Z, Virgin H, Kyei GB, Johansen T, Vergne I, Deretic V. 2010. Delivery of cytosolic components by autophagic adaptor protein p62 endows autophagosomes with unique antimicrobial properties. Immunity 32:329–341. doi:10.1016/j.immuni.2010.02.009. 20206555 PMC2846977

[78] Alonso S, Pethe K, Russell DG, Purdy GE. 2007. Lysosomal killing of *Mycobacterium* mediated by ubiquitin-derived peptides is enhanced by autophagy. Proc Natl Acad Sci USA 104:6031–6036. doi:10.1073/pnas.0700036104. 17389386 PMC1851611

[79] Altan N, Chen Y, Schindler M, Simon SM. 1999. Tamoxifen inhibits acidification in cells independent of the estrogen receptor. Proc Natl Acad Sci USA 96:4432–4437. doi:10.1073/pnas.96.8.4432. 10200279 PMC16349

[80] Chen Y, Schindler M, Simon SM. 1999. A mechanism for tamoxifen-mediated inhibition of acidification. J Biol Chem 274:18364–18373. doi:10.1074/jbc.274.26.18364. 10373441

[81] Lu SY, Sung T, Lin NW, Abraham RT, Jessen BA. 2017. Lysosomal adaptation: how cells respond to lysosomotropic compounds. PLoS One 12:e0173771. doi:10.1371/journal.pone.0173771. 28301521 PMC5354416

[82] Song TT, Cai RS, Hu R, Xu YS, Qi BN, Xiong YA. 2021. The important role of TFEB in autophagy-lysosomal pathway and autophagy-related diseases: a systematic review. Eur Rev Med Pharmacol Sci 25:1641–1649. doi:10.26355/eurrev_202102_24875. 33629334

[83] Zewdie KA, Hailu HG, Ayza MA, Tesfaye BA. 2022. Antileishmanial activity of tamoxifen by targeting sphingolipid metabolism: a review. Clin Pharmacol 14:11–17. doi:10.2147/CPAA.S344268. 35221731 PMC8880078

[84] Sfogliarini C, Pepe G, Dolce A, Della Torre S, Cesta MC, Allegretti M, Locati M, Vegeto E. 2022. Tamoxifen twists again: on and off-targets in macrophages and infections. Front Pharmacol 13:879020. doi:10.3389/fphar.2022.879020. 35431927 PMC9006819

[85] Meeker ND, Hutchinson SA, Ho L, Trede NS. 2007. Method for isolation of PCR-ready genomic DNA from zebrafish tissues. Biotechniques 43:610, 612, 614. 18072590 10.2144/000112619

[86] van der Sar AM, Abdallah AM, Sparrius M, Reinders E, Vandenbroucke-Grauls CM, Bitter W. 2004. *Mycobacterium marinum* strains can be divided into two distinct types based on genetic diversity and virulence. Infect Immun 72:6306–6312. doi:10.1128/IAI.72.11.6306-6312.2004. 15501758 PMC523024

[87] Takaki K, Davis JM, Winglee K, Ramakrishnan L. 2013. Evaluation of the pathogenesis and treatment of *Mycobacterium marinum* infection in zebrafish. Nat Protoc 8:1114–1124. doi:10.1038/nprot.2013.068. 23680983 PMC3919459

[88] Benard EL, van der Sar AM, Ellett F, Lieschke GJ, Spaink HP, Meijer AH. 2012. Infection of zebrafish embryos with intracellular bacterial pathogens. J Vis Exp doi:10.3791/3781. PMC3415172 22453760

[89] Stoop EJM, Schipper T, Huber SKR, Nezhinsky AE, Verbeek FJ, Gurcha SS, Besra GS, Vandenbroucke-Grauls CMJE, Bitter W, van der Sar AM. 2011. Zebrafish embryo screen for mycobacterial genes involved in the initiation of granuloma formation reveals a newly identified ESX-1 component. Dis Model Mech 4:526–536. doi:10.1242/dmm.006676. 21372049 PMC3124061

[90] Schindelin J, Arganda-Carreras I, Frise E, Kaynig V, Longair M, Pietzsch T, Preibisch S, Rueden C, Saalfeld S, Schmid B, Tinevez JY, White DJ, Hartenstein V, Eliceiri K, Tomancak P, Cardona A. 2012. Fiji: an open-source platform for biological-image analysis. Nat Methods 9:676–682. doi:10.1038/nmeth.2019. 22743772 PMC3855844

[91] McQuin C, Goodman A, Chernyshev V, Kamentsky L, Cimini BA, Karhohs KW, Doan M, Ding L, Rafelski SM, Thirstrup D, Wiegraebe W, Singh S, Becker T, Caicedo JC, Carpenter AE. 2018. CellProfiler 3.0: next-generation image processing for biology. PLoS Biol 16:e2005970. doi:10.1371/journal.pbio.2005970. 29969450 PMC6029841

[92] Xie Y, Tolmeijer S, Oskam JM, Tonkens T, Meijer AH, Schaaf MJM. 2019. Glucocorticoids inhibit macrophage differentiation towards a pro-inflammatory phenotype upon wounding without affecting their migration. Dis Model Mech 12:dmm037887. doi:10.1242/dmm.037887. 31072958 PMC6550045

[93] Patro R, Duggal G, Love MI, Irizarry RA, Kingsford C. 2017. Salmon provides fast and bias-aware quantification of transcript expression. Nat Methods 14:417–419. doi:10.1038/nmeth.4197. 28263959 PMC5600148

[94] RStudio Team. 2020. RStudio: integrated development for R. RStudio. http://www.rstudio.com/. Accessed 28 April 2021.

[95] R Core Team. 2018. R: a language and environment for statistical computing. R Foundation for Statistical Computing, Vienna, Austria.

[96] Soneson C, Love MI, Robinson MD. 2015. Differential analyses for RNA-seq: transcript-level estimates improve gene-level inferences. F1000Res 4:1521. doi:10.12688/f1000research.7563.1. 26925227 PMC4712774

[97] Love MI, Huber W, Anders S. 2014. Moderated estimation of fold change and dispersion for RNA-seq data with DESeq2. Genome Biol 15:550. doi:10.1186/s13059-014-0550-8. 25516281 PMC4302049

[98] Zhu A, Ibrahim JG, Love MI. 2019. Heavy-tailed prior distributions for sequence count data: removing the noise and preserving large differences. Bioinformatics 35:2084–2092. doi:10.1093/bioinformatics/bty895. 30395178 PMC6581436

[99] Stephens M. 2017. False discovery rates: a new deal. Biostatistics 18:275–294. doi:10.1093/biostatistics/kxw041. 27756721 PMC5379932

[100] Allen MA-O, Poggiali DA-O, Whitaker KA-O, Marshall TR, Kievit RA-O. 2019. Raincloud plots: a multi-platform tool for robust data visualization. Wellcome Open Res 4:63. doi:10.12688/wellcomeopenres.15191.1. 31069261 PMC6480976

