# Supplementary material for: Repurposing Tamoxifen as Potential Host-Directed Therapeutic for Tuberculosis
Source: mBio. 2022 Dec 7;14(1):e03024-22. doi: 10.1128/mbio.03024-22 (PMC9973281; doi:10.1128/mbio.03024-22)
Supplement: TABLE S1 [file mbio.03024-22-st001.pdf]

Supplementary table S1: CFU data of Mϕ1 and Mϕ2 corresponding to Figure 1.

Figure 1A												
	**MΦ1**						**MΦ2**					
	**CTRL**			**TAM**			**CTRL**			**TAM**		
Donor	Average	Stdev	N	Average	Stdev	N	Average	Stdev	N	Average	Stdev	N
1	171667	51153	6	190000	10000	3	286667	85010	6	280000	105830	3
2	215000	28810	6	223333	23094	3	238333	79854	6	70000	36056	3
3	237500	83890	6	201250	88729	4	96667	39455	6	53750	50724	4
4	140833	44656	6	85000	27988	4	151667	42269	6	26250	17017	4
5	410000	97018	5	160000	39051	3	755000	196373	5	500000	43301	3
6	408000	109579	5	188333	57951	3	312000	108317	5	160000	35000	3
7	203333	61455	6	130000	55000	3	202500	76207	6	193333	35473	3
8	131667	104921	3	103333	20207	3	231667	94851	6	165000	91924	2

Figure 1B												
	**MΦ1**						**MΦ2**					
	**CTRL**			**TAM**			**CTRL**			**TAM**		
Donor	Average	Stdev	N	Average	Stdev	N	Average	Stdev	N	Average	Stdev	N
1	14000	1000	3	633	231	3	12300	4782	3	6600	8359	3
2	42000	1414	2	867	115	3	29667	9292	3	133	115	3
3	15333	8083	3	933	586	3	21333	4163	3	3133	2053	3
4	17667	2517	3	1533	577	3	12000	3464	3	467	208	3
5	26667	1528	3	1833	1955	3	36667	8622	3	3200	2615	3
6	20667	8505	3	2967	306	3	17667	5508	3	1000	361	3
