# Supplementary figures and images for: Repurposing Tamoxifen as Potential Host-Directed Therapeutic for Tuberculosis
Source: mBio. 2022 Dec 7;14(1):e03024-22. doi: 10.1128/mbio.03024-22 (PMC9973281; doi:10.1128/mbio.03024-22)

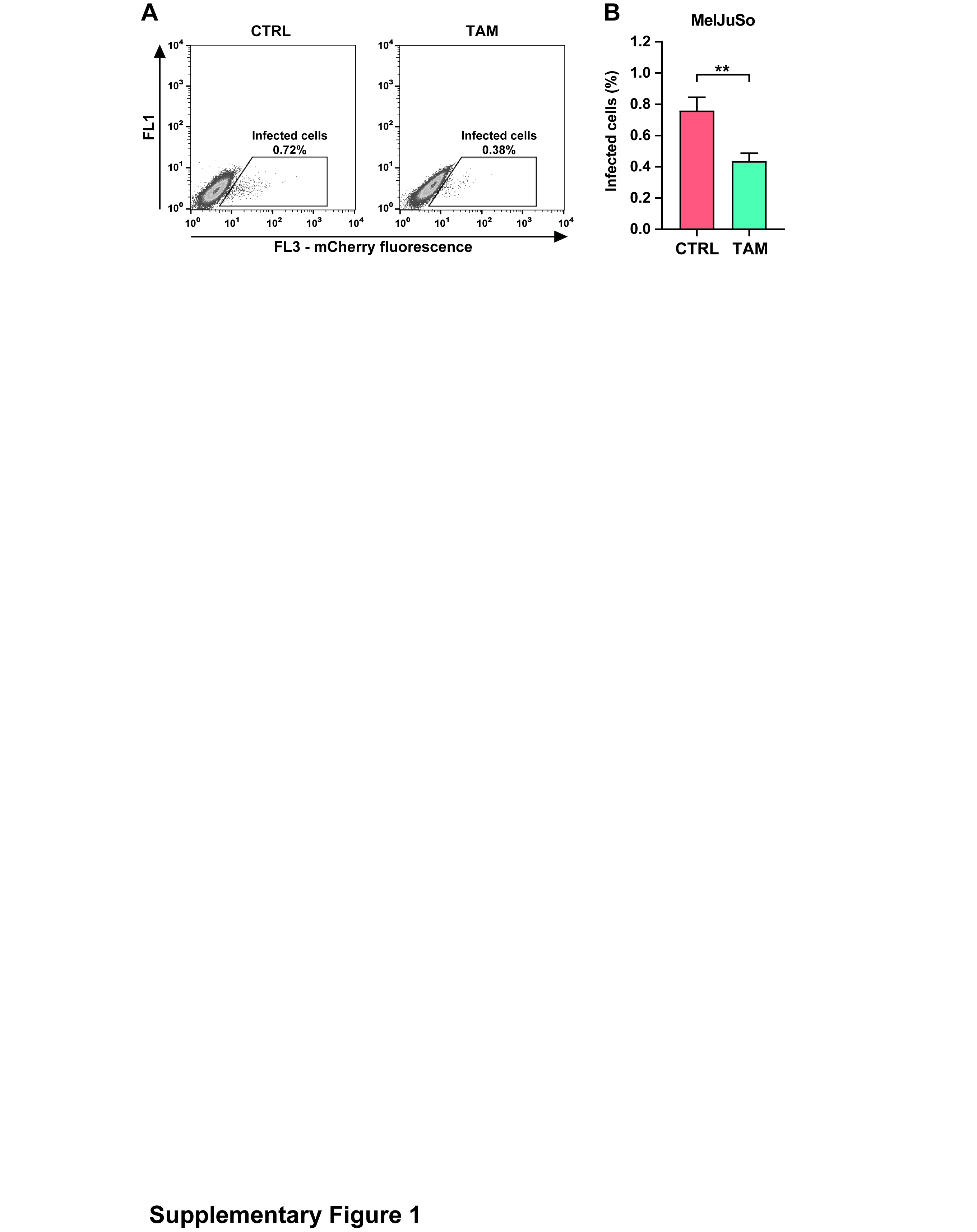

Supplement: FIG S1 [file mbio.03024-22-sf001.tif]

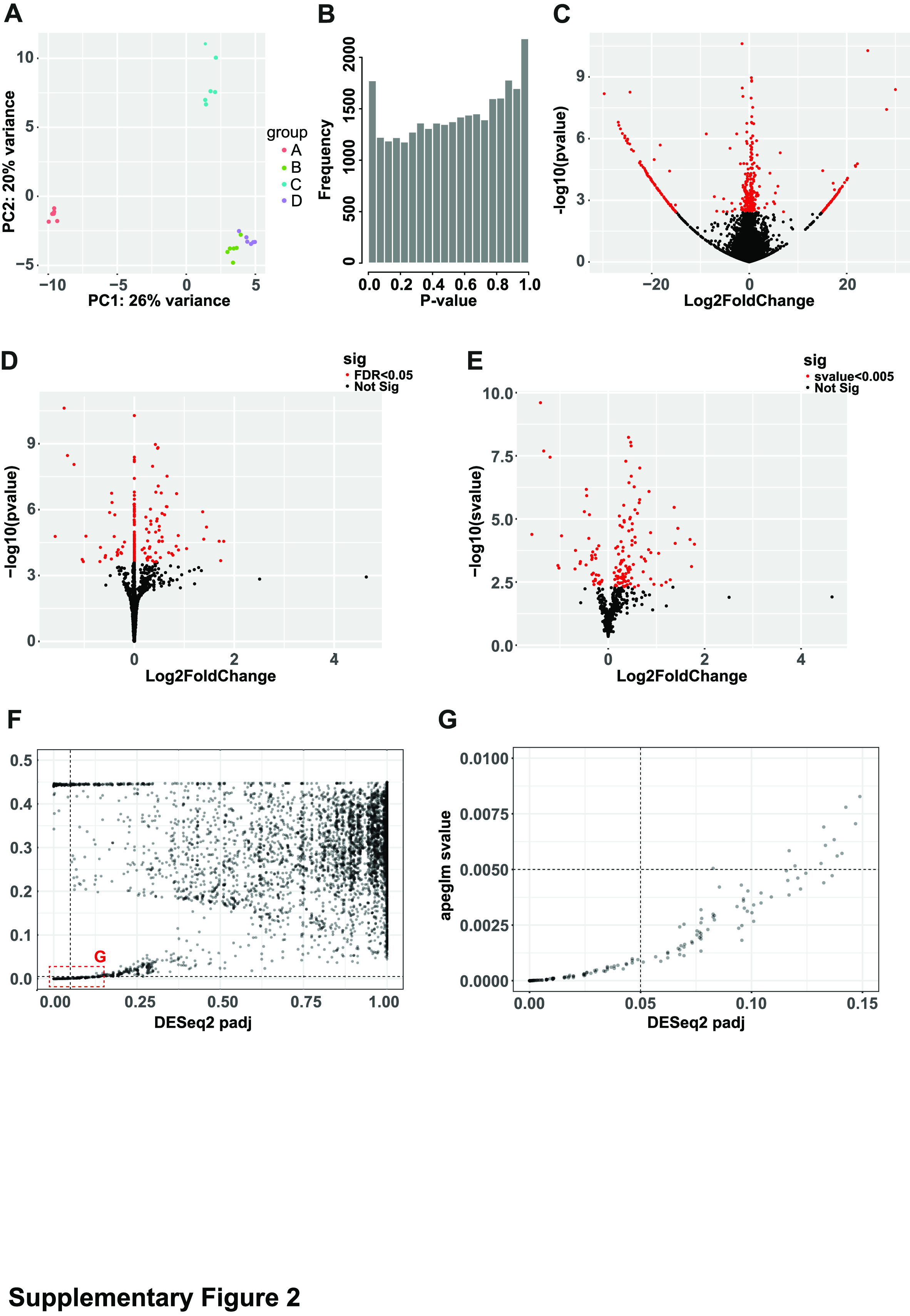

Supplement: FIG S2 [file mbio.03024-22-sf002.tif]

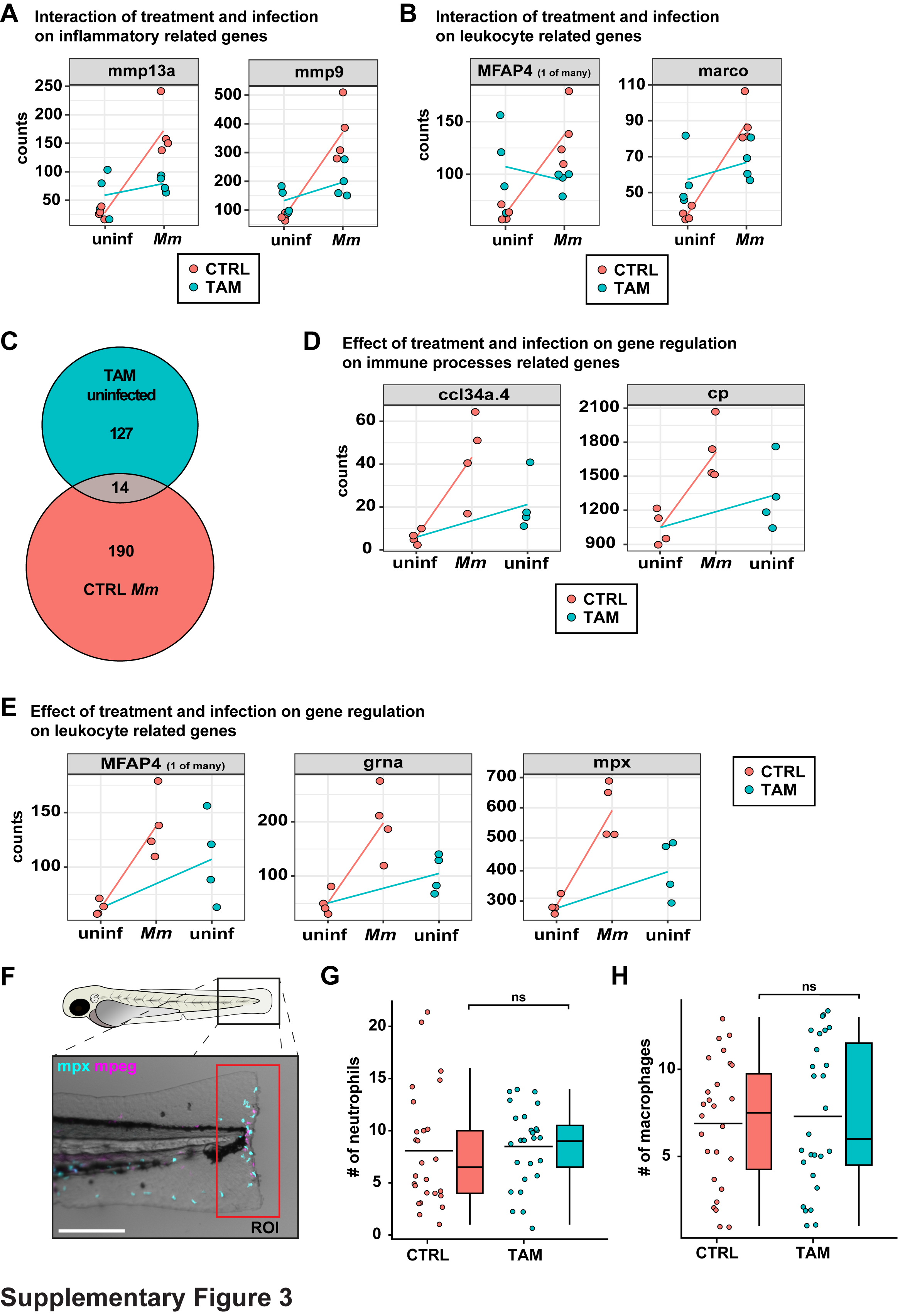

Supplement: FIG S3 [file mbio.03024-22-sf003.tif]

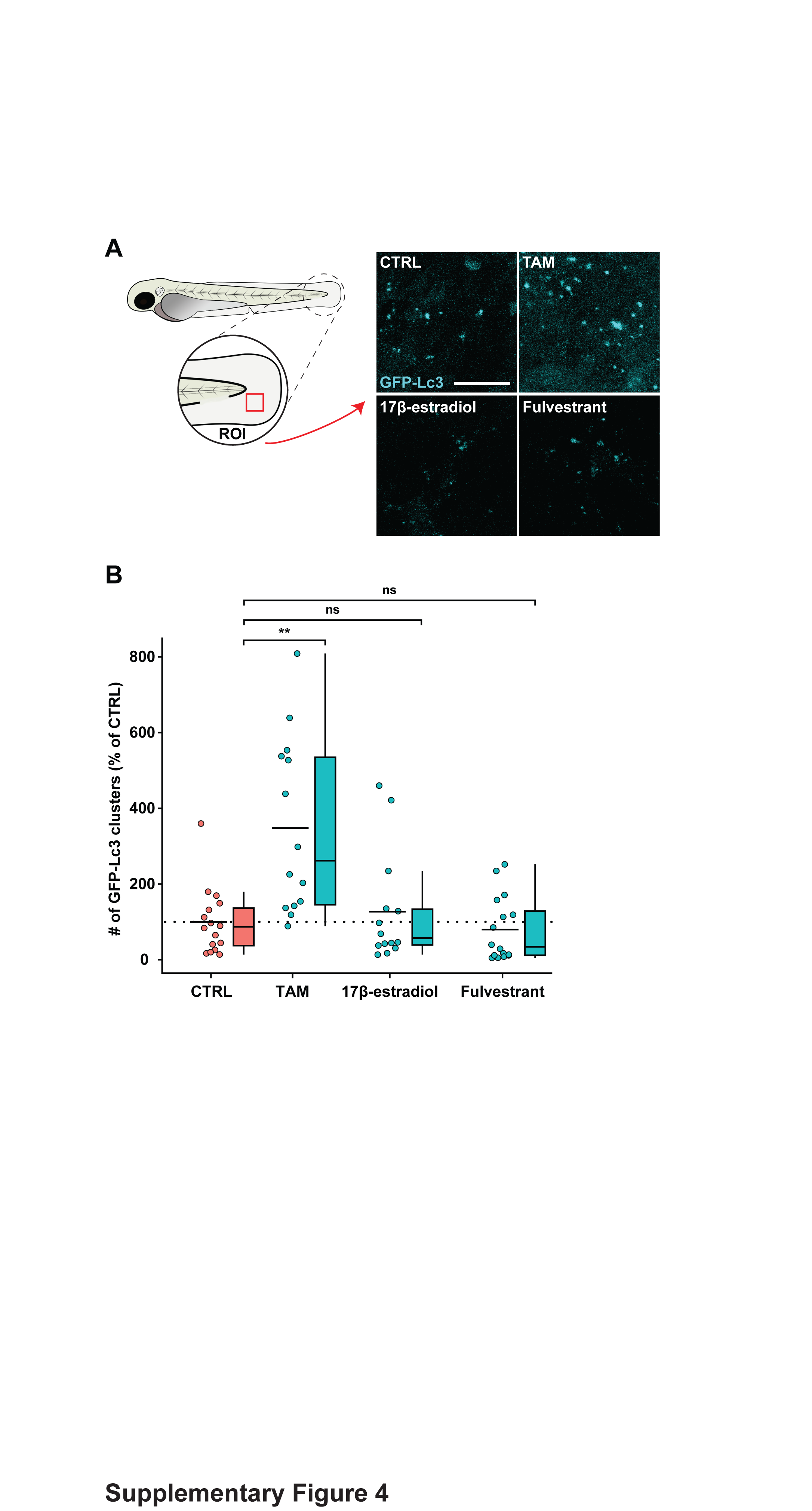

Supplement: FIG S4 [file mbio.03024-22-sf004.tif]
