# Supplementary material for: Repurposing Tamoxifen as Potential Host-Directed Therapeutic for Tuberculosis
Source: mBio. 2022 Dec 7;14(1):e03024-22. doi: 10.1128/mbio.03024-22 (PMC9973281; doi:10.1128/mbio.03024-22)
Supplement: TABLE S2 [file mbio.03024-22-st002.pdf]

**Supplementary table S2: Interaction of treatment and infection on gene regulation**

| Gene name | Ensembl ID | s-value | Log2FC |
|---|---|---|---|
| AL935146.1 | ENSDARG00000112812 | 0,003884 | 0,521512122 |
| amd1 | ENSDARG00000043856 | 0,000341 | 0,242002141 |
| BX005175.1 | ENSDARG00000112442 | 7,37E-05 | -0,670979432 |
| c3a.6 | ENSDARG00000043719 | 0,002685 | -0,545814275 |
| c4b | ENSDARG00000038424 | 0,000121 | -0,696489422 |
| cnot2 | ENSDARG00000061802 | 0,003033 | -0,281112148 |
| CT573383.1 | ENSDARG00000097513 | 0,00213 | -0,789054804 |
| epg5 | ENSDARG00000059846 | 0,001415 | -0,438146512 |
| FERMT3 (1 of many) | ENSDARG00000079267 | 0,004768 | -1,181817384 |
| gdi2 | ENSDARG00000113039 | 0,0007 | -0,294281106 |
| hck | ENSDARG00000058647 | 0,000785 | -0,732845923 |
| hmcn2 | ENSDARG00000079327 | 0,001891 | -0,37352186 |
| itgb2 | ENSDARG00000016939 | 0,000284 | -0,704094034 |
| marco | ENSDARG00000059294 | 0,000603 | -0,846209025 |
| MFAP4 (1 of many) | ENSDARG00000088745 | 0,000214 | -1,071839201 |
| mfsd13a | ENSDARG00000112339 | 0,000522 | -1,160824835 |
| mmp13a | ENSDARG00000012395 | 7,33E-06 | -1,905795573 |
| mmp9 | ENSDARG00000042816 | 1,95E-05 | -1,589634402 |
| musk | ENSDARG00000098764 | 0,001222 | -0,637124638 |
| psmc4 | ENSDARG00000027099 | 0,000902 | -0,507563082 |
| ptpn13 | ENSDARG00000103699 | 0,00347 | -0,494978538 |
| rasal2 | ENSDARG00000036257 | 0,001658 | -0,526200288 |
| ric8b | ENSDARG00000005972 | 0,004342 | -0,581914895 |
| rmc1 | ENSDARG00000029307 | 0,000445 | -1,856523793 |
| si:ch211-147m6.1 | ENSDARG00000109648 | 4,43E-10 | -1,378532336 |
| si:ch211-194m7.3 | ENSDARG00000074322 | 0,000388 | -0,844262364 |
| si:dkey-88l16.2 | ENSDARG00000095137 | 0,002367 | -2,336116068 |
| trim63a | ENSDARG00000111657 | 0,001029 | 1,039071884 |
