# Supplementary material for: Repurposing Tamoxifen as Potential Host-Directed Therapeutic for Tuberculosis
Source: mBio. 2022 Dec 7;14(1):e03024-22. doi: 10.1128/mbio.03024-22 (PMC9973281; doi:10.1128/mbio.03024-22)
Supplement: TABLE S3 [file mbio.03024-22-st003.pdf]

Supplementary table S3: Effect of treatment and infection on gene regulation

Gene name	Ensembl ID	s-value (CTRL)	Log2FC (CTRL)	s-value (TAM)	Log2FC (TAM)
BX005175.1	ENSDARG00000101334	0,001093545	0,364449917	2,64E-05	0,495096771
ccl34a.4	ENSDARG00000074656	3,49E-11	2,834446032	6,52E-05	1,697390717
cfhl4	ENSDARG00000010312	0,001131535	0,218548149	0,000362287	-0,256087622
cp	ENSDARG00000090873	2,55E-13	0,687125852	0,003422273	0,257557489
ctsc	ENSDARG00000116951	9,22E-07	0,582920465	9,62E-08	0,65480504
ctss2.1	ENSDARG00000113068	3,89E-09	1,35242256	0,001239314	0,725429688
cul1a	ENSDARG00000004954	0,000260209	1,20735414	9,29E-05	1,389402743
grna	ENSDARG00000112150	1,59E-10	1,908385972	0,00237872	0,880257892
hist2h2l	ENSDARG00000019521	0,000665246	0,373924602	0,004626593	0,310629696
MFAP4 (1 of many)	ENSDARG00000112442	2,93E-07	1,031906608	0,004953604	0,545848653
mpx	ENSDARG00000109648	5,49E-13	1,058634459	0,002816962	0,417152838
si:ch211-147m6.1	ENSDARG00000105142	2,04E-46	2,342056078	8,18E-07	0,845930154
tcirg1b	ENSDARG00000006019	1,52E-08	0,548631879	0,002258719	0,284884932
tktb	ENSDARG00000088745	0,001875833	0,173521655	0,000247625	0,214566765
