# Supplementary material for: Repurposing Tamoxifen as Potential Host-Directed Therapeutic for Tuberculosis
Source: mBio. 2022 Dec 7;14(1):e03024-22. doi: 10.1128/mbio.03024-22 (PMC9973281; doi:10.1128/mbio.03024-22)
Supplement: TABLE S4 [file mbio.03024-22-st004.pdf]

Supplementary table S4: KEGG pathway and Gene Ontology (GoSeq) analysis

KEGG pathway		
**Pathway**	**Number of DR genes**	**padj**
Lysosome	16 (out of 140)	5,88E-14
Other glycan degradation	4 (out of 24)	0,00108355
Apoptosis	7 (out of 164)	0,003841255
Phagosome	6 (out of 142)	0,010176479
Glycosphingolipid biosynthesis - globo and isoglobo series	2 (out of 8)	0,028298257
Metabolic pathways	18 (out of 1286)	0,028298257
Ferroptosis	3 (out of 41)	0,048131463
Autophagy - animal	5 (out of 154)	0,05438032
mTOR signaling pathway	5 (out of 181)	0,09609386

Gene Ontology (GoSeq)				
**GO term**	**Category**	**Ontology**	**Number of DR genes**	**p-adj**
hydrolase activity	GO:0016787	MF	29 (out of 1268)	1,34E-07
peptidase activity	GO:0008233	MF	14 (out of 435)	0,00012964
proteolysis	GO:0006508	BP	18 (out of 752)	0,00012964
lysosome	GO:0005764	CC	7 (out of 74)	0,00012964
cysteine-type peptidase activity	GO:0008234	MF	9 (out of 147)	0,00012964
hydrolase activity, acting on glycosyl bonds	GO:0016798	MF	6 (out of 74)	0,004329959
metabolic process	GO:0008152	BP	6 (out of 82)	0,007120488
carbohydrate metabolic process	GO:0005975	BP	8 (out of 208)	0,013229959
lysosomal membrane	GO:0005765	CC	4 (out of 46)	0,078774283
