# Supplementary material for: Repurposing Tamoxifen as Potential Host-Directed Therapeutic for Tuberculosis
Source: mBio. 2022 Dec 7;14(1):e03024-22. doi: 10.1128/mbio.03024-22 (PMC9973281; doi:10.1128/mbio.03024-22)
Supplement: TABLE S5 [file mbio.03024-22-st005.pdf]

Supplementary table S5: Supplementary materials

**Zebrafish lines**		
**Name**	**Description**	**Reference**
AB/TL	Wild type strain	Zfin.org
esr2b^{sa77}	Loss of function *esr2b* mutant	Lopez-Munoz 2015
Tg(CMV:EGFP-map1lc3b)^{zf155}	GFP-tagged zebrafish Lc3	He 2009
Tg(mpeg1:mCherryF)^{umsF001}	Macrophage marker	Bernut 2014
Tg(mpx:EGFP)^{i114}	Neutrophil marker	Renshaw 2006
Tg(mpeg1:mCherryF, mpx:EGFP)$^{umsF001, i114}$	Macrophage and neutrophil marker	Bernut 2014, Renshaw 2006

**Primer sequences**				
**Gene**	**Type**	**Ensemble ID**	**Sequence**	
esr2b	PCR*	ENSDARG00000034181	FW	TCTTGGATGACATTAATAATCTGG
			RV	ATTCAACTGCAGTGTCTTGC
tbp	qPCR	ENSDARG00000014994	FW	CCTGCCCATTTTCAGTC
			RV	TGTTGTTGCCTCTGTTGCTC
cyp19a1b	qPCR	ENSDARG00000098360	FW	AAAGAGTTACTAATAAAGATCCACCGGTAT
			RV	TCCACAAGCTTTCCCATTTCA
vtg1	qPCR	ENSDARG00000092233	FW	ACTACCAACTGGCTGCTTAC
			RV	ACCATCGGCACAGATCTTC

* The esr2b forward primer was also used for sequencing.
